# Estimates of the Burden of Group B Streptococcal Disease Worldwide for Pregnant Women, Stillbirths, and Children

**DOI:** 10.1093/cid/cix664

**Published:** 2017-11-06

**Authors:** Anna C Seale, Fiorella Bianchi-Jassir, Neal J Russell, Maya Kohli-Lynch, Cally J Tann, Jenny Hall, Lola Madrid, Hannah Blencowe, Simon Cousens, Carol J Baker, Linda Bartlett, Clare Cutland, Michael G Gravett, Paul T Heath, Margaret Ip, Kirsty Le Doare, Shabir A Madhi, Craig E Rubens, Samir K Saha, Stephanie J Schrag, Ajoke Sobanjo-ter Meulen, Johan Vekemans, Joy E Lawn

**Affiliations:** 1 Maternal, Adolescent, Reproductive and Child Health Centre, London School of Hygiene & Tropical Medicine, United Kingdom;; 2 College of Health and Medical Sciences, Haramaya University, Dire Dawa, Ethiopia;; 3 King’s College London, United Kingdom;; 4 Centre for Child and Adolescent Health, School of Social and Community Medicine, University of Bristol, United Kingdom;; 5 Neonatal Medicine, University College London Hospitals NHS Foundation Trust, United Kingdom;; 6 Department of Reproductive Health Research, University College London Institute for Women’s Health, United Kingdom;; 7 ISGlobal, Barcelona Centre for International Health Research, Hospital Clinic, University of Barcelona, Spain;; 8 Departments of Pediatrics and Molecular Virology and Microbiology, Baylor College of Medicine, Houston, Texas;; 9 Department of International Health, Johns Hopkins Bloomberg School of Public Health, Baltimore, Maryland;; 10 Medical Research Council: Respiratory and Meningeal Pathogens Research Unit, and Department of Science and Technology/National Research Foundation: Vaccine Preventable Diseases, Faculty of Health Sciences, University of the Witwatersrand,Johannesburg, South Africa;; 11 Global Alliance to Prevent Prematurity and Stillbirth, Seattle, Washington;; 12 Department of Obstetrics and Gynecology, University of Washington School of Medicine, Seattle;; 13 Vaccine Institute, Institute for Infection and Immunity, St George’s University of London and St George’s University Hospitals NHS Foundation Trust, United Kingdom;; 14 Department of Microbiology, Faculty of Medicine, Chinese University of Hong Kong;; 15 Centre for International Child Health, Imperial College London, United Kingdom;; 16 National Institute for Communicable Diseases, National Health Laboratory Service, Johannesburg, South Africa;; 17 Department of Global Health, University of Washington, Seattle;; 18 Bangladesh Institute of Child Health, Dhaka;; 19 National Center for Immunization and Respiratory Diseases, Centers for Disease Control and Prevention, Atlanta, Georgia;; 20 Bill & Melinda Gates Foundation, Seattle, Washington; and; 21 World Health Organization, Geneva, Switzerland

**Keywords:** group B *Streptococcus*, infection, newborn, stillbirth, maternal

## Abstract

**Background:**

We aimed to provide the first comprehensive estimates of the burden of group B *Streptococcus* (GBS), including invasive disease in pregnant and postpartum women, fetal infection/stillbirth, and infants. Intrapartum antibiotic prophylaxis is the current mainstay of prevention, reducing early-onset infant disease in high-income contexts. Maternal GBS vaccines are in development.

**Methods:**

For 2015 live births, we used a compartmental model to estimate (1) exposure to maternal GBS colonization, (2) cases of infant invasive GBS disease, (3) deaths, and (4) disabilities. We applied incidence or prevalence data to estimate cases of maternal and fetal infection/stillbirth, and infants with invasive GBS disease presenting with neonatal encephalopathy. We applied risk ratios to estimate numbers of preterm births attributable to GBS. Uncertainty was also estimated.

**Results:**

Worldwide in 2015, we estimated 205000 (uncertainty range [UR], 101000–327000) infants with early-onset disease and 114000 (UR, 44000–326000) with late-onset disease, of whom a minimum of 7000 (UR, 0–19000) presented with neonatal encephalopathy. There were 90000 (UR, 36000–169000) deaths in infants <3 months age, and, at least 10000 (UR, 3000–27000) children with disability each year. There were 33000 (UR, 13000–52000) cases of invasive GBS disease in pregnant or postpartum women, and 57000 (UR, 12000–104000) fetal infections/stillbirths. Up to 3.5 million preterm births may be attributable to GBS. Africa accounted for 54% of estimated cases and 65% of all fetal/infant deaths. A maternal vaccine with 80% efficacy and 90% coverage could prevent 107000 (UR, 20000–198000) stillbirths and infant deaths.

**Conclusions:**

Our conservative estimates suggest that GBS is a leading contributor to adverse maternal and newborn outcomes, with at least 409000 (UR, 144000–573000) maternal/fetal/infant cases and 147000 (UR, 47000–273000) stillbirths and infant deaths annually. An effective GBS vaccine could reduce disease in the mother, the fetus, and the infant.

## Teaser

Group B *Streptococcus* is an important cause of disease in pregnant women, stillbirth, and infants. These first estimates show the magnitude and the potential impact of maternal vaccination.

The number of worldwide child deaths has declined, from an estimated 12.7 million in 1990 to 5.9 million in 2015 [1]. However, there has been less progress in reducing neonatal mortality and stillbirths, with 2.7 million neonatal deaths and 2.6 million stillbirths in 2015 [2, 3]. Maternal mortality remains unacceptably high, with an estimated 303000 deaths in 2015. Most of this burden is in low-income settings, particularly in sub-Saharan Africa and South Asia [1, 2, 4].

Infection is an important cause of maternal, fetal, and infant mortality in low- and middle-income contexts [1, 5–7]. However, in addition to the substantial burden of mortality, there is a mostly unquantified burden of infection-related short- and long-term morbidity [8]. Infections are also an important underlying contributor to preterm birth and neonatal encephalopathy, which, along with infections, are leading causes of neonatal mortality and subsequent adverse outcomes worldwide [8–11].

Understanding of specific infectious etiologies is, however, limited [12]. Quantifying the burden of individual etiologies is necessary to inform public health interventions. Group B *Streptococcus* (GBS) is an important perinatal pathogen [13, 14], yet to date no systematic estimates have been undertaken of its overall global burden [15].

GBS is a frequent colonizer of the maternal gastrointestinal and genital tracts. Overall, 18% (95% confidence interval [CI], 17%–19%) of women worldwide are estimated to be colonized, although there is regional variation in prevalence, ranging from a high prevalence in the Caribbean of 35% (95% CI, 35%–40%), to a much lower prevalence in Southern Asia and Eastern Asia (13% [95% CI, 10%–14%] and 11% [95% CI, 10%–12%], respectively) [16]. Ascending infection can cause maternal, fetal, and early-onset neonatal disease (days 0–6), leading to maternal death, stillbirth, and/or neonatal death [17–19]. In survivors of neonatal or young infant GBS disease, neurodevelopmental impairment may result [20]. In addition to causing invasive neonatal disease, maternal GBS colonization also increases the risk of preterm birth [21]. Neonatal encephalopathy (NE) may occur with invasive GBS disease, but maternal GBS colonization and ascending infection also increases the risk of NE [22].

Preventive measures aimed at reducing the risk of invasive early-onset GBS disease (EOGBS) in newborns have focused on intrapartum antibiotic prophylaxis (IAP), with intravenous antibiotics given to women in labor, based either on microbiological screening or clinical risk factors [23]. However, this depends on national policy and a health system with the capacity to implement either strategy with appropriate coverage. While reductions in EOGBS disease (days 0–6 after birth) in the United States have been observed [24], IAP does not prevent late-onset GBS disease (LOGBS; days 7–89) [25] and is unlikely to have an impact on stillbirth or preterm birth. GBS vaccines are in development [26] and, if given to women, could be effective in preventing these outcomes as well as infant and maternal invasive GBS disease [15]. Vaccine candidates include protein-based formulations and serotype-specific polysaccharide-protein conjugates [27] and thus an understanding of serotype distribution in maternal and infant disease worldwide is important.

**Figure 1.**
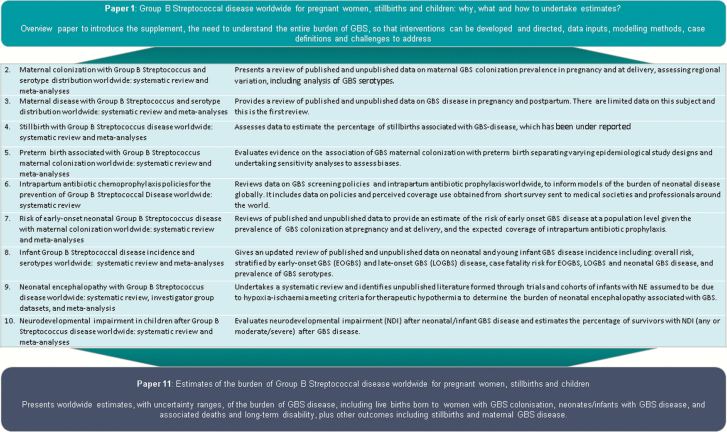
Overview of the articles in this supplement to estimate the worldwide burden of group B *Streptococcus.* Adapted from Lawn et al [15]. Abbreviations: GBS, group B *Streptococcus*; NE, neonatal encephalopathy.

**Figure 2.**
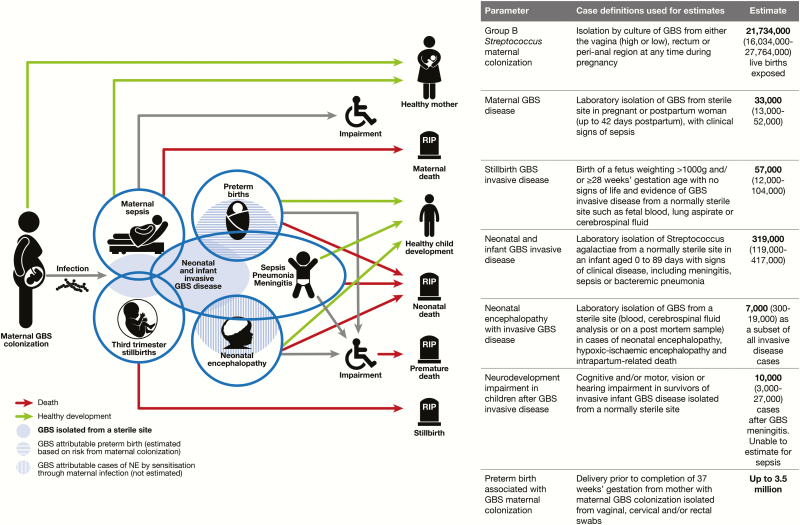
Disease schema for outcomes of maternal group B *Streptococcus* colonization showing worldwide estimates for 2015. Adapted from Lawn et al [15]. Abbreviations: GBS, group B *Streptococcus*; NE, neonatal encephalopathy.

**Figure 3.**
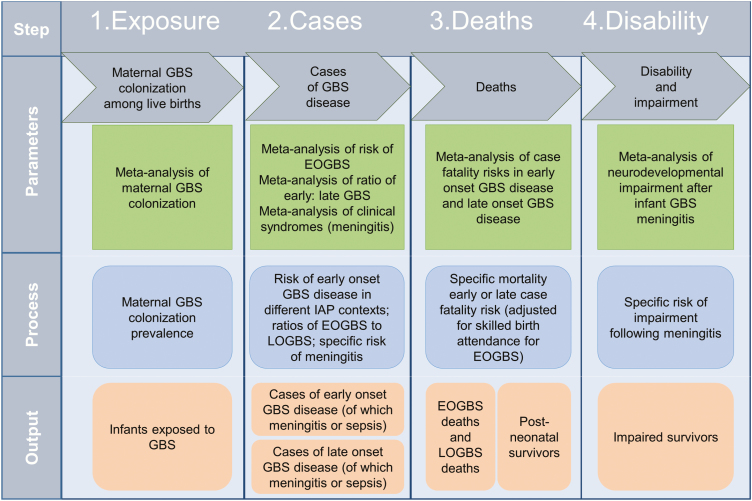
Compartmental model for estimating cases of infant group B streptococcal disease, deaths, and disability. Abbreviations: EOGBS, early-onset group B *Streptococcus*; GBS, group B *Streptococcus*; IAP, intrapartum antibiotic prophylaxis; LOGBS, late-onset group B *Streptococcus*.

This is the last article in a supplement estimating the burden of invasive GBS disease in pregnant and postpartum women, stillbirths, and infants (Figure 1) [15]. The supplement includes systematic reviews and meta-analyses across the disease burden schema (Figure 2). These provide input parameters into the compartmental model described here, for infant GBS cases, deaths, and disability (Figure 3). We also estimate maternal GBS disease, stillbirths with GBS disease, the subset of cases of infant GBS disease who also have neonatal encephalopathy, and preterm birth attributable to GBS. These are reported according to international guidelines [28, 29].

## OBJECTIVES

We aimed to:

1. Estimate national, regional, and worldwide numbers of infants in 2015 with invasive GBS disease (including those presenting with neonatal encephalopathy), and outcomes in terms of deaths and disability, using a compartmental model.
2. Estimate national, regional, and worldwide numbers of cases in 2015, using pooled estimates of incidence, proportions or risk ratios, derived from meta-analyses for:
  a. maternal GBS disease,
  b. stillbirths with invasive GBS disease, and
  c. Preterm birth attributed to maternal GBS colonization.
3. Estimate the number of maternal and infant cases, infant deaths, and stillbirths currently prevented by IAP, and preventable cases and deaths with high worldwide IAP coverage and/or maternal GBS vaccination.
4. Describe GBS serotypes colonizing mothers and causing maternal and infant GBS disease, summarizing reported regional variation.

## METHODS

We summarize our methods according to our 4 objectives as follows:

1. Estimate national, regional, and worldwide numbers of infants in 2015 with invasive GBS disease (including those presenting with neonatal encephalopathy) and outcomes in terms of deaths and disability, using a compartmental model.

### Modeling Approach

We conceptualized the full burden of GBS disease (Figure 2) to include pregnant and postpartum women, fetal infections (based on stillbirths), and infants, as described in the first article in this supplement [15]. We took a compartmental model approach to modeling infant invasive GBS disease, deaths, and disability, with 4 steps as illustrated in Figure 3. For the first step in the model (maternal GBS colonization), the step where most data were available for national prevalence estimation, we also attempted a multivariable regression model to predict national maternal GBS colonization, as an alternative to using a subregional estimate when national-level data were limited (Appendix).

### Data Inputs

We sought data inputs from the published literature through systematic reviews and unpublished sources through research databases and investigators worldwide, as summarized in the previous 10 articles (Figure 1). The specific methods used for each of these (database searches, inclusion and exclusion criteria, data characteristics, criteria used to assess bias and sensitivity analyses) are described in general [15] and reported elsewhere [16–23, 30]. We performed meta-analyses, to obtain estimates of maternal GBS colonization prevalence [16], the ratio of late-onset to early-onset invasive GBS disease [19], case fatality risks (CFRs) [19], proportion of cases with meningitis [19], proportion of infants with GBS meningitis who had moderate to severe neurodevelopmental impairment [20], incidence of maternal GBS disease in pregnant/postpartum women [17], prevalence of GBS disease in stillbirth [18], prevalence of GBS disease in neonatal encephalopathy [22], and the association between maternal GBS colonization and preterm birth [21]. We calculated pooled estimates using random-effects models [31] to allow for heterogeneity across studies by use of a statistical parameter representing the variation between studies.

### Burden Estimation Applying the Compartmental Model

#### Step 1. Exposure to Maternal Group B Streptococcus Colonization

For the first step of the compartmental model, we determined maternal GBS colonization prevalence for countries, subregions (South America, Central America, Caribbean, Western Asia, Southern Asia, South-Eastern Asia, Eastern Asia, Oceania) and regions (Latin America, Asia, Africa, Oceania, developed) as described elsewhere [16], to apply to estimates of live births in 195 countries for 2015, using latest United Nations data [32]. The colonization data were adjusted for sampling site (rectal and/or vaginal) and laboratory culture methods [16]. Where data were considered sufficient (≥1000 mothers tested for rectovaginal colonization), we used an estimate for individual countries. Where data were limited (<1000 mothers tested for rectovaginal colonization), we used a subregional estimate, and where no subregional estimate was available, we used a regional estimate (Supplementary Table 1 for inputs by country).

#### Step 2. Cases of Invasive Early-Onset Disease and Late-Onset Disease in Different Intrapartum Antibiotic Prophylaxis Settings

For the second step of the compartmental model, we assessed IAP policies and their implementation in countries as described elsewhere in this supplement [23], and categorized 89 countries with data available into 1 of 4 categories, which were (1) microbiological screening for maternal GBS colonization with IAP and high implementation coverage (>50% of mothers screened and given IAP if appropriate); (2) clinical risk factor approach with IAP given to mothers with risk factors before delivery and high implementation coverage (>50% with risk factors receiving IAP); (3) microbiological screening for maternal GBS colonization with IAP and low implementation coverage (<50%); (4) clinical risk factor approach with IAP given to mothers with risk factors before delivery and low implementation coverage (<50%), or no IAP strategy in place. We assigned countries in the developed region with no data to category 1 as a conservative approach, and of those countries reporting these data, 21 of 31 developed countries were in group 1. We assigned countries, not in the developed region and with no data to group 4, as 51 of 59 countries not in the developed region reporting these data were in this group.

We then assessed the risk of EOGBS disease in studies reporting maternal GBS colonization data, and the use of IAP, as described elsewhere in this supplement [30]. We used the linear association between IAP use and risk of EOGBS disease described in [23] to estimate the risk of EOGBS disease in each of the 4 contexts, with specific risks for each group as follows: group 1 = 0.3% (95% CI, .0–.9%); group 2 = 0.6% (95% CI, .10%–1.2%); group 3 = 0.9% (95% CI, .4%–1.5%); group 4 = 1.1% (95% CI, .6%–1.5%). For each country, the number of cases of EOGBS was estimated by multiplying the estimated number of exposed babies by the appropriate risk for that country.

We used regional estimates of the ratio of early-onset to late-onset GBS cases [19] to then estimate the number of LOGBS cases. For Oceania, where data were lacking, we applied the estimate for Asia, as the most similar regional context. There were variations in estimates, with the highest ratio in Asia (5.99 [95% CI, 2.40–14.9]) suggesting more EOGBS than LOGBS, and lowest in Africa (1.02 [95% CI, 0.82–1.27]). We give parameters for each region in Table 1. These regional estimates could, however, be affected by low case ascertainment. This could reduce EOGBS disease cases, particularly those with home delivery, inadequate access to care and/or high rapid CFR, and/or late-onset cases, particularly if cerebrospinal fluid sampling is not undertaken, and cases of GBS meningitis are thus not detected. We therefore did a sensitivity analysis applying a worldwide ratio of early-onset to late-onset GBS disease from high-quality studies worldwide (1.11 [95% CI, 0.90–1.30] / 3.92) [19].

#### *Step 3. Deaths in Early-Onset* *and Late-Onset Group B* Streptococcus *Disease*

For the third step of the compartmental model, we applied region-specific CFRs to 3 different groups that differ considerably in terms of outcome: EOGBS cases delivered without a skilled birth attendant, EOGBS cases delivered with a skilled birth attendant, and LOGBS cases.

*Case fatality risk for EOGBS:* We applied percentages of skilled birth attendance for each country to EOGBS cases to determine EOGBS cases which would, and would not, have been attended by a skilled birth attendant. We applied a CFR of 0.9 (0.3–1.0) to estimated EOGBS cases born without a skilled birth attendant, based on expert opinion as to the likely high CFR in these “unseen” cases. To estimate deaths from EOGBS born with a skilled birth attendant (and for all developed countries), we estimated regional CFRs for EOGBS from facility-based data, as described elsewhere in this supplement [19]. We applied these regional CFRs to cases of EOGBS disease with skilled birth attendance. The highest CFR for EOGBS with skilled attendance was in Africa (0.27 [0.15–0.37]), then Latin America (0.17 [0.05–0.30]), Asia (0.14 [0.06–0.23]), and developed countries (0.05 [0.04–0.07]) (Table 1). For Oceania, where even regional data were lacking, we applied the risk in Asia, being the most geographically proximal.

*Case fatality risk for LOGBS:* We also estimated regional CFRs for LOGBS from facility-based data, as described elsewhere in this supplement [19]. Regional CFRs for LOGBS were lower than EOGBS overall, with the highest again in Africa (0.12 [0.05–0.19]) (Table 1). Due to insufficient data from Oceania, we applied the CFR for Asia.

**Table 1.** Data Inputs to the Compartmental Model to Estimate Cases of Infant Group B Streptococcal Disease, Deaths, and Disability

			Asia					Africa					Oceania	Latin America and Caribbean			
			Southern Asia	Eastern Asia	Central Asia	West Asia	SE Asia	Northern Africa	Southern Africa	Eastern Africa	Western Africa	Mid. Africa	Oceania	Caribbean	Central America	South America	Developed
No. of countries			9	4	5	18	11	6	5	18	16	9	14	13	8	12	47
No. of live births in 2015			37M	18M	1.6M	5.8M	12.3M	6.1M	1.2M	13.9M	13.4M	6.0M	0.03M	0.7M	0.3M	7.0M	13.4M
Step 1																	
	Percentage of infants exposed to maternal GBS at birth [16]	Countries	4	3	0	5	4	2	1	4	3	2	1		1	11	21
		Datasets	44	41		32	14	8	7	22	18	3	1	5	6	35	83
		Pregnant women	15838	63289		15124	3591	1576	13218	14071	4860	2058	440	1137	3229	16141	144604
			12.5 (10.2–14.8)	11.1 (9.9– 12.4)		14.7 (12.1–17.4)	14.4 (11.5–17.4)	22.9 (17.0–28.2)	28.9 (26.6–31.2)	19.4 (15.9–23.0)	17.5 (10.8–24.1)	23.9 (14.7–33.1)		34.7 (29.5–39.9)	17.1 (13.2–21.0)	18.4 (15.5–21.3)	19.2 [17.7–20.7]
Step2	IAP policy [23]	Countries	28					20						11			31
		IAP group where known	Group 2 = 1; group 3 = 1; group 4 = 26					Group 2 = 1; group 4 = 19						Group 1 = 4; group 3 = 1; group 4 = 6			Group 1 = 21; group 2 = 7; group 4 = 3
		Datasets	14 (from varying IAP policy contexts in 8 countries)														
		GBS cases	85														
		By IAP policy	Group 1 = 0.003 (0.0–0.009); group 2 = 0.006 (0.001–0.012); group 3 = 0.009 (0.004–0.015); group 4 = 0.011 (0.006–0.015)														
	Ratio of EOGBS to LOGBS [30]	Countries	3					6						3			12
		Datasets	4					7						3			13
		GBS cases	123					1352						50			3217
			5.60 (2.40–14.9)					1.02 (0.82–1.07)						1.90 (0.98–3.69)			1.82 (1.29–2.57)
	Proportion of meningitis cases in EOGBS [19]	Countries	30														
		Datasets	26														
		Meningitis cases	176														
			0.12 (0.08–0.15)														
	Proportion of meningitis cases in [19]	Countries	17														
		Datasets	18														
		Meningitis cases	689														
			0.42 (0.30–0.55)														
Step 3	Case fatality risk in EOGBS without skilled birth attendance 0.9 (0.3–1.0) estimated																
	Case fatality risk (proportion) in EOGBS in a facility [30]	Countries	8					5						7			18
		Datasets	12					6						9			19
		Deaths	36					131						15			123
			0.14 (0.06–0.23)					0.27 (0.15–0.37)						0.17 (0.05–0.30)			0.05 (0.04–0.07)
	Case fatality risk (proportion) of LOGBS [30]	Countries	3					5						3			14
		Datasets	6					5						14			3
		Deaths	12					116						3			67
			0.05 (0.02–0.09)					0.12 (0.05–0.19)						0.06 (0–0.19)			0.04 (0.03–0.06)
Step 4	NDI risk (proportion) in infant meningitis (EOGBS and LOGBS) [20]	Countries															4
		Datasets															12
		Cases															80
																	0.18 (0.13–0.22)

Data in parentheses represent the 95% confidence interval.

Abbreviations: EOGBS, early-onset group B *Streptococcus*; GBS, group B *Streptococcus*; IAP, intrapartum antibiotic prophylaxis; LOGBS, late-onset group B *Streptococcus*; NDI, neurodevelopmental impairment.

#### *Step 4. Disability or Impairment* *After Infant Group B* Streptococcus *Meningitis*

We estimated moderate to severe neurodevelopmental impairment (NDI) after meningitis, only, because data were insufficient to estimate NDI after sepsis, as described elsewhere in this supplement [20]. To do this, we applied the percentage of infant cases of GBS disease which were meningitis, for early (12% [8%–15%]) and late-onset (42% [30%–55%]) GBS disease [18] to estimates of EOGBS and LOGBS survivors. We then applied an incidence risk of moderate to severe NDI at 18 months of age of 0.18 (0.13–0.22) [20]. These data were limited to developed countries; however, we applied this proportion worldwide, on the basis that this would be a minimum estimate as NDI was unlikely to be lower in settings with reduced levels of care.

### Triangulation of Infant Invasive Group B *Streptococcus* Disease Cases From the Compartmental Model With Estimates Based on Incidence Data

We compared the results from the compartmental model for infant GBS disease cases with those estimated using incidence data on infant GBS disease [19]. To do this, we calculated subregional incidence, or regional incidence where subregional data were not available, of EOGBS and LOGBS disease. We applied these to estimates of live births for each country in 2015. Data inputs are given for each country in Supplementary Table 2.

### Infants With Invasive Group B *Streptococcus* Disease Presenting With Neonatal Encephalopathy

To calculate the numbers of infants with invasive GBS disease and coexistent neonatal encephalopathy, we used previously published national incidences of neonatal encephalopathy and modeled uncertainties and adjusted these for births in 2015 [11]. Then using our new data, we calculated the proportion of invasive GBS disease among these cases of NE. In developed countries, among all NE cases included in cooling trials, 0.51% (95% CIs, 0.05%–0.97%) were also identified as having GBS disease [22]. Data inputs were limited for data from other regions (3/16 studies), so we used the worldwide estimate of 0.58% (95% CIs, 0.18%–0.98%) of NE cases with GBS disease to apply in Africa, Asia, Latin America, and Oceania. Since our case definition assumes that cases of NE with GBS count as a case of GBS invasive disease, we include these numbers within our estimates of GBS infant disease.

2. Estimate country, regional, and worldwide number of cases of GBS-associated maternal disease, stillbirths, and preterm birth, for births in 2015 using pooled estimates of incidence, proportions, or risk ratios, derived from meta-analyses.

Where a compartmental approach was not possible, we used incidence, prevalence, or risk ratios from pooled data applied to births in 2015 to make minimum estimates of worldwide, regional, and national estimates for cases attributable to GBS (Figure 2).

a. Maternal GBS disease

We calculated the pooled incidence of maternal GBS disease per 1000 maternities and applied this to a denominator of total births worldwide to estimate cases. As described elsewhere [17], data were only available for developed countries, with a pooled estimate of 0.23 (95% CI, .09–.37) per 1000 maternities. We applied this to all regions, on the basis that maternal GBS disease was unlikely to be lower in settings with reduced levels of care.

b. Stillbirths with GBS disease

We calculated the pooled prevalence of GBS disease in stillbirths, equating also to the minimum number of fetal infections. Data were available from developed countries (1% [95% CIs, 0–2%]) and from Africa (4% [95% CIs, 2%–6%]) [18]. For regions with no data, we applied the prevalence of GBS in stillbirths from developed countries, on the basis that GBS-associated stillbirth was unlikely to be lower in settings with reduced levels of care. However, as this is a conservative approach, we did a sensitivity analysis applying the regional estimate from Africa (4% [95% CIs, 2%–6%]) [18] to regions with no data.

c) Preterm birth associated with maternal GBS colonization

We calculated pooled risk ratios or odds ratios for the association between maternal GBS colonization and preterm birth [21]. For cohort or cross-sectional studies, the risk ratio was 1.21 (95% CI, .99–1.48; *P* = .061), and for case-control studies, the odds ratio was 1.85 (95% CI, 1.24–2.77; *P* = .003). However, for preterm birth the results, in terms of the association between maternal colonization and preterm birth, are susceptible to confounding and bias. For preterm birth, we thus give a range for the number of cases, based on calculation of the population attributable fraction, which could be attributable to GBS given maternal GBS colonization [16] and incidence of preterm birth [33]. The ranges are based on the range in the 95% CIs of risk and odds ratios (1.0–2.8) for the association between maternal GBS colonization and preterm birth.

3. Estimate maternal and infant cases, stillbirths, and infant deaths, prevented by IAP at present, and preventable cases and deaths with high worldwide IAP coverage and/or maternal GBS vaccination.

We applied risks, without adjusting for IAP use, to estimates of live births for 2015 to calculate early-onset cases with no IAP use. We adjusted for skilled birth attendance as previously and applied regional facility CFRs to estimate deaths with no IAP use. We subtracted current cases and deaths in early infancy to calculate those currently prevented by IAP.

For IAP scale-up worldwide, we assumed that all births were being attended by a skilled birth attendant, able to provide careful clinical monitoring for risk factors at delivery and administer IAP, but we did not adjust CFRs for this. Given these assumptions, we applied risks of EOGBS disease with a clinical risk factor approach, with coverage >50% and IAP worldwide where microbiological screening and IAP was not already in place. We did not calculate cases prevented with IAP for pregnant or postpartum women or stillbirths, or late-onset cases as these are not the target of IAP and any effect is likely to be limited due to the timing of IAP administration.

For maternal GBS vaccination, we calculated cases prevented (with no IAP) by a maternal GBS vaccine with 80% efficacy and coverage at 50% and 90%, for births in 2015. No assumptions were made on skilled birth attendance and/or laboratory capacity.

4. Describe GBS serotypes colonizing mothers and causing maternal and infant GBS disease.

We calculated the prevalence of GBS serotypes (Ia/Ib/II–X) colonizing mothers and causing maternal and infant GBS disease from meta-analyses of proportions of each serotype reported in each disease syndrome [16, 17, 19]. We calculated the coverage of a pentavalent maternal GBS vaccine (Ia/Ib/II/III/V) based on these data.

### Uncertainty Estimation

For the compartmental model, we included uncertainty at every step by taking 1000 random draws, assuming a normal distribution with a mean equal to the point estimate of the parameter, and standard deviation (SD) equal to the estimated standard error (SE) of the parameter. We present the 2.5th and 97.5th centiles of the resulting distributions as the uncertainty range (UR).

For the incidence or proportional approach, we estimated uncertainty around the point estimate with the same approach, taking 1000 random draws, assuming a normal distribution with a mean equal to the point estimate of the parameter, and SD equal to the estimated SE of the parameter. We present the 2.5th and 97.5th centiles of the resulting distributions as the UR.

### Source Code

Code used for the estimation process is available online at https://doi.org/10.17037/data.51.

## RESULTS

We summarize our results according to our 4 objectives as follows:

1. Estimate country, regional, and worldwide cases of invasive infant GBS disease, and outcomes in terms of deaths and disabilities for live births in 2015 using a compartmental model.

### Step 1. Exposure to Maternal Group B *Streptococcus* Colonization

We estimated that, of 140 million live births in 2015, there were 21.3 million (UR, 16.4–27.0 million) infants exposed to maternal GBS colonization at delivery. There were 74.5 million live births in Asia with 8.9 million (UR, 6.7–10.7 million) infants exposed, 40.7 million live births in Africa with 8.0 million (UR, 5.3–10.3 million) infants exposed, 11.0 million live births in Latin America with 2.1 million (UR, 1.7–2.5 million) infants exposed, 260000 live births in Oceania with 33000 (UR, 31–36000) infants exposed and 13.4 million live births in developed countries with 2.8 million (UR, 2.3–3.2 million) infants exposed (subregional estimates in Supplementary Figure 3).

### Step 2. Cases of Early-Onset and Late-Onset Disease in Different Intrapartum Antibiotic Prophylaxis Settings

**Figure 4.**
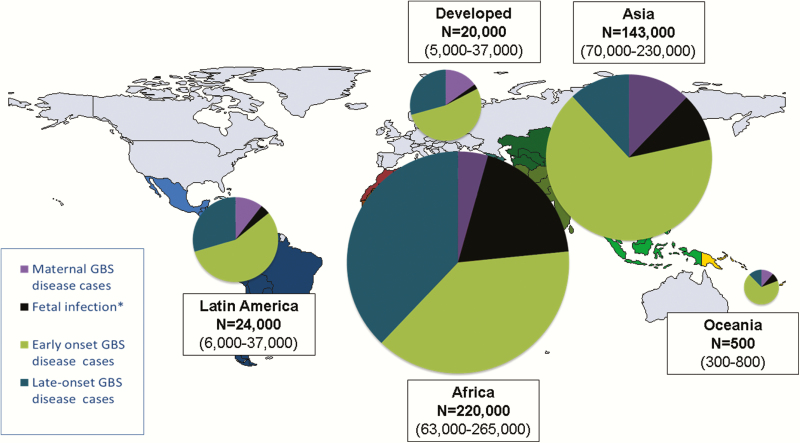
Cases estimated for group B streptococcal (GBS) disease in pregnant/postpartum women, fetuses, and infants in 2015, by United Nations Sustainable Development Goal region. *Stillbirths represent a minimum estimate of fetal infection cases. More details are shown in Supplementary Figures 4, 11, and 12.

We estimated that there were 319000 cases (UR, 119000–417000) of infant invasive GBS disease worldwide. Most cases were EOGBS disease, with 205000 (UR, 101000–327000) cases compared to 114000 (UR, 44000–326000) LOGBS cases. With a high absolute number of births, and thus newborns exposed, Asia had the highest number of EOGBS disease cases, with 95000 (UR, 53–143000). Africa had fewer EOGBS cases 85000 (UR, 44–133000), but, because of the differences in early-onset to late-onset disease ratios, more LOGBS disease cases, with 84000 (UR, 43–140000) in Africa compared to 17000 (UR, 0–146000) in Asia. In contrast, developed countries had 11000 (UR, 0–26000) cases of EOGBS and 6000 (UR, 0–15000) cases of LOGBS disease (Table 2; Figure 4; Supplementary Figure 4). Using a fixed worldwide ratio of early-onset to late-onset disease based only on high-quality studies (sensitivity analysis), we estimated a higher 184000 (UR, 142–196000) LOGBS infant cases. Asia accounted for this increase, with 84000 (UR, 65–90000) LOGBS disease cases (Supplementary Figure 5).

**Table 2.** Estimated Cases of Maternal, Fetal, and Infant Group B Streptococcal Disease in 2015

Region	Maternal GBS Disease	Fetal Infection^a^	EOGBS Disease	LOGBS Disease
Southern Asia	8700	9700	42500	7600
	(4000–14000)	(1200–21300)	(23000–65400)	(0–57000)
Eastern Asia	4100	1300	21900	3900
	(1700–6700)	(0–2300)	(12700–32900)	(0–30000)
Central Asia	400	200	2300	400
	(200–600)	(0–400)	(1300–3200)	(0–3000)
Western Asia	1300	800	9200	1600
	(600–2200)	(0–1700)	(5100–13800)	(0–12400)
South-Eastern Asia	2900	1500	19400	3500
	(1200–4600)	(0–3300)	(10800—28700)	(0–43200)
Asia	35900	13400	95300	17000
	(7100–28100)	(1200–29600)	(52800–142900)	(0–145600)
Oceania	60	40	400	100
	(20–100)	(0–100)	(10800–28700)	(0–43100)
Northern Africa	1400	3900	15400	15000
	(600–2300)	(1000–6700)	(8600–22400)	(8300–24000)
Southern Africa	300	800	4000	3900
	(100–500)	(200–1400)	(2300–5500)	(2300–6000)
Eastern Africa	3300	12600	26400	25900
	(1300–4600)	(3100–21700)	15300–40300)	14700–42700)
Western Africa	3200	18300	23500	23000
	(1300–5200)	(4500–30800)	(10200–39000)	(10300–41000)
Middle Africa	1400	6300	15900	15600
	(3300–12500)	(1600–10800)	(7900–25600)	(7500–25900)
Africa	9600	42000	85200	20700
	(6700–25000)	(10400–71400)	(44300–132800)	(43100–140000)
Caribbean	200	100	2600	1300
	(60–300)	(0–200)	(1500–3700)	(0–4300)
Central America	800	200	3100	1700
	(300–1300)	(1700–13400)	(200–6430)	(0–6100)
South America	1600	600	8000	4200
	(700–2600)	(0–1200)	(1700–14400)	4000–14500)
Latin America	3700	900	13700	5900
	(1000–4100)	(0–2000)	(3400–24400)	(4000–24800)
Developed countries	3000	500	10900	6000
	(1300–5000)	(0–800)	(0–25800)	(0–15500)
Total	32800	56800	205500	113800
	(13400–52100)	(11600–103900)	(44200–326000)	(0–11100)

Data in parentheses represent the uncertainty range (UR).

Abbreviations: EOGBS, early-onset group B Streptococcus; GBS, group B Streptococcus; IAP, intrapartum antibiotic prophylaxis; LOGBS, late-onset group B Streptococcus; NDI, neurodevelopmental impairment.

^a^Stillbirths indicated a minimum estimate of cases of fetal infection.

### Step 3. Deaths in Early-Onset and Late-Onset Group B *Streptococcus* Disease

We estimated that there were 90000 (UR, 36000–169000) deaths in infants due to invasive GBS disease worldwide. Africa accounted for 54000 (UR, 22000–98000) of these, Asia 31000 (UR, 13000–60000), Latin America 4000 (600–10000), Oceania 200 (UR, 60–300), and developed countries 800 (UR, 0–2000).

**Figure 5.**
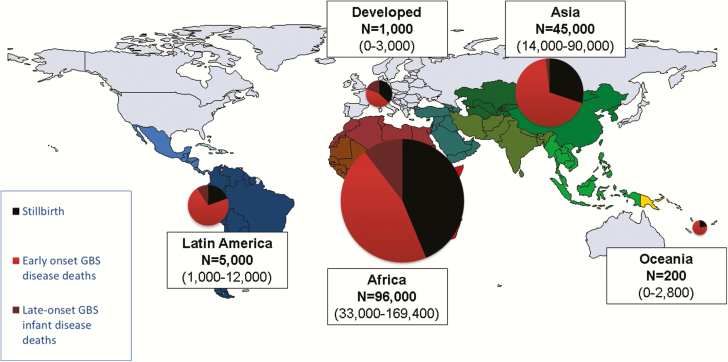
Deaths estimated from group B streptococcal (GBS) disease for infants and stillbirths in 2015, by United Nations Sustainable Development Goal region. Maternal deaths not estimated. More details are shown in Supplementary Figures 6 and 12.

In terms of deaths due to EOGBS, there were 51000 deaths (UR, 23000–89000) in infants without access to healthcare worldwide. There were a further 27000 (UR, 9000–50000) deaths from EOGBS in facilities in developing countries. In contrast, there were 500 (UR, 0–1300) deaths in developed countries from EOGBS. In terms of LOGBS deaths, overall deaths were lower, with 12000 (UR, 3–30000) worldwide. Most of these 10000 (UR, 3000–21000) were in Africa. Regional and subregional estimates are given in Table 2 and Supplementary Figure 6 and illustrated in Figure 5. Countries with the highest number of cases are not always those with the highest number of deaths, as illustrated for Nigeria, Ethiopia, and Pakistan (Table 3).

**Table 3.** Countries With the Highest Estimated Numbers of Infant Group B Streptococcal Disease Cases and Deaths

Cases			Deaths		
Rank	Country	Infant Cases	Rank	Country	Infant Deaths
1	India	31000	1	India	13000
		(0–75000)			(5000–23000)
2	China	25 000	2	Nigeria	8000
		(0–59000)			(2000–16000)
3	Nigeria	22000	3	Ethiopia	4000
		(8000–39000)			(2000–8000)
4	Democratic Republic of the Congo	16000	4	Democratic Republic of the Congo	4000
		(8000–39000)			(2000–7000)
5	Egypt	14000	5	Pakistan	3000
		(8000–21000)			(1000–6000)

Data in parentheses represent the uncertainty range.

### Step 4. Disability: Calculation of Impairment After Infant Group B *Streptococcus* Meningitis

We estimated that a minimum of 10000 (UR, 3000–27000) infants worldwide had moderate to severe NDI after GBS meningitis. Of these, more than half were in Africa (6000 [UR, 3000–12000]), with 3000 (UR, 0–11000) in Asia, 700 (UR, 100–2300) in Latin America, 700 (UR, 0–1700) in developed countries, and <100 (UR, 0–100) in Oceania (Table 4; Supplementary Figure 7).

**Table 4.** Stillbirth, Infant Deaths From Group B Streptococcal Disease and Resultant Disability Estimated in 2015

Region	Stillbirth	Early Infant Deaths	Late Infant Deaths	Disability
Southern Asia	9700	19600	400	1000
	(1200–21300)	(8500–34400)	(0–2800)	(0–4500)
Eastern Asia	1300	3200	200	700
	(0–2800)	(1100–5800)	(0–1600)	(0–2400)
Central Asia	200	400	0	100
	(0–400)	(100–600)	(0–200)	(0–300)
Western Asia	800	2100	100	300
	(0–1700)	(900–10900)	(0–700)	(0–1100)
South-Eastern Asia	1500	5200	200	600
	(0–3300)	(2100–8900)	(0–1400)	(0–2300)
Asia	13400	30400	900	2600
	(1200–29600)	(12700–60600)	(0–6600)	(0–10600)
Oceania	40	100	0	10
	(0–90)	(60–200)	(0–30)	(0–40)
Northern Africa	4000	6600	1800	1200
	(1000–6700)	(3100–10900)	(600–3600)	(600–2100)
Southern Africa	800	1200	500	300
	(200–1400)	(600–1900)	(100–900)	(200–500)
Eastern Africa	12600	15600	3100	2000
	(3100–21700)	(7500–26800)	(1000–6400)	(1000–3600)
Western Africa	18300	13400	2800	1800
	(4500–30800)	(5000–24500)	(800–6000)	(700–3300)
Middle Africa	6300	7300	1900	1200
	(1500–10800)	(3100–12600)	(600–3900)	(500–2400)
Africa	42000	44000	10000	6400
	(10400–71400)	(19200–76700)	(3100–20800)	(3000–11900)
Caribbean	100	900	100	100
	(0–200)	(300–1600)	(0–400)	(60–400)
Central America	200	800	100	200
	(0–500)	(100–2000)	(0–600)	(0–600)
South America	600	1600	300	400
	(0–1200)	(1400–3700)	(0–1400)	(60–1300)
Latin America	900	3300	400	700
	(0–2000)	(1900–7200)	(0–2400)	(100–2300)
Developed countries	500	500	200	700
	(0–800)	(0–1300)	(0–700)	(0–1700)
Total	56800	78400	11500	10500
	(11600–103900)	(32500–138900)	(3100–30500)	(3000–26000)

Data are presented as estimate (uncertainty range).

#### Triangulation of Infant Invasive Group B Streptococcus Disease Cases From the Compartmental Model With Estimates Based on Incidence Data

**Figure 6.**
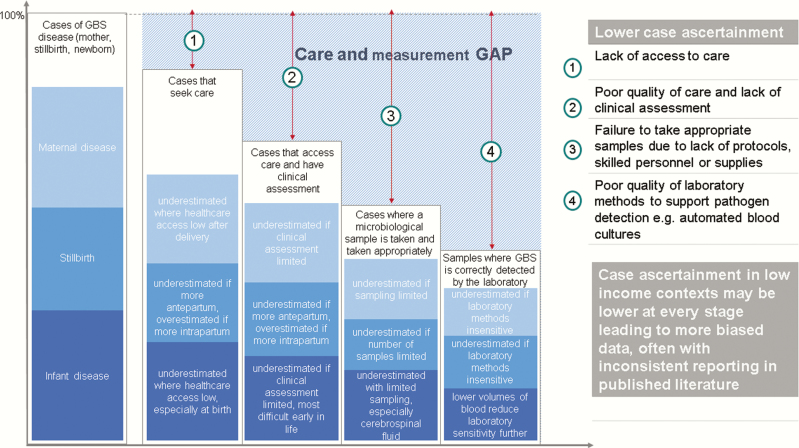
Care and measurement gap estimating cases from incidence and prevalence data. Adapted from Lawn et al [15]. Triangulation of estimates from compartmental model compared to incidence data for invasive infant disease is detailed in Supplementary Figures 8 and 9. Abbreviation: GBS, group B *Streptococcus*.

Applying pooled incidences of EOGBS and LOGBS to the 140 million live births for 2015, we estimated a much lower burden, particularly for EOGBS cases, than that estimated using the compartmental model. We estimated 51000 (UR, 23000–89000) infants with EOGBS and 40000 (UR, 12000–75000) infants with LOGBS worldwide (subregional estimates in Supplementary Figures 8 and 9). These are likely to be considerable underestimates as cases are systematically underascertained, particularly in low- and middle-income contexts, as described in Table 2 and Figure 6.

### Infants With Invasive Group B *Streptococcus* Disease Presenting With Neonatal Encephalopathy

We estimated that there were a minimum of 7000 (300–19000) infants with invasive GBS disease presenting with neonatal encephalopathy. There were an estimated 3400 (UR, 200–9000) cases in Asia, 3300 (UR, 100–8600) in Africa, 300 (UR, 0–1200) in Latin America, 100 (UR, 0–300) in developed countries, and 10 (UR, 0–40) in Oceania (subregional estimates are given in Supplementary Figure 10).

2. Estimate country, regional, and worldwide cases, for births in 2015 using pooled estimates of incidence, proportions, or risk ratios, derived from meta-analyses for maternal GBS disease, stillbirth with GBS disease, and preterm birth associated with maternal GBS colonization:
  a. Maternal GBS disease

We estimated that there were a minimum of 33000 (UR, 13–52000) cases of maternal invasive GBS disease worldwide. Estimates are given by subregion in Supplementary Figure 11 and region in Table 4.

b. Stillbirth with GBS disease

We estimated that there were a minimum of 57000 (UR, 12000–104000) cases of stillbirth with GBS disease worldwide, equating to a minimum of 57000 (UR, 12000–104000) fetal infections. Of these, Africa accounted for 42000 (UR, 10000–71000) and Asia 13000 (UR, 1000–30000) (Supplementary Figure 12 and Table 4). Applying the higher regional estimate for Africa to regions where there are no data (sensitivity analysis), the number of stillbirths with GBS disease was much higher, at 96000 (UR, 26–168000) worldwide, with Asia accounting for 50000 (UR, 14000–87000) of these, almost all the increase (Supplementary Figure 13).

c. Preterm birth attributable to GBS

We estimated that the range of cases of preterm birth attributable to GBS was 0–3.5 million. The cases of preterm birth attributable to GBS according to each risk ratio (in 0.2 increments [1.0–2.8]) are given in Supplementary Table 4.

3. Estimate maternal and infant cases, infant deaths, and stillbirths prevented by IAP at present, and preventable cases and deaths with high worldwide IAP coverage and/or maternal GBS vaccination.

**Figure 7.**
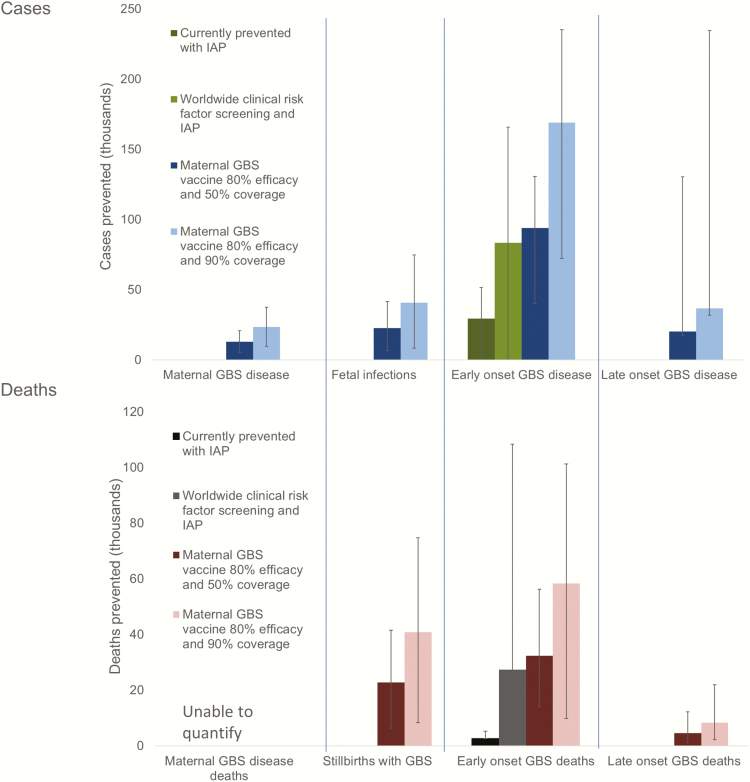
Scenarios of estimated cases of group B streptococcal (GBS) disease and deaths prevented with different intervention methods in a year. For worldwide clinical risk factor screening and intrapartum antibiotic prophylaxis (IAP) where microbiological screening was in place, this estimate was applied for that country. To facilitate comparison between the current situation and interventions, case fatality risks have been applied as at present (ie, a higher case fatality risk for deliveries without a skilled birth attendant).

Contingent in the limitations in our estimates, we estimated that 29000 infants (UR, 0–51000) with EOGBS and 3000 (UR, 0–108000) infant deaths were prevented by intrapartum antibiotic prophylaxis worldwide in 2015. With worldwide application of a clinical risk factor–based approach (microbiological screening where already in place), and IAP (>50% coverage), we estimate that 83000 (UR, 0–166000) cases of EOGBS and 27000 (UR, 0–110000) deaths could be prevented worldwide (not adjusting CFRs for the changes in skilled birth attendance that IAP administration would require). With worldwide maternal vaccination (and no IAP assumed), a maternal GBS vaccine with 80% efficacy and 50% coverage would prevent 127000 (UR, 63000–282000) infant and maternal GBS cases, 23000 (UR, 6000–42000) stillbirths, and 37000 (UR, 15000–68000) infant deaths. A maternal vaccine with the same assumptions with 90% coverage would prevent 229000 (UR, 114000–507000) infant and maternal GBS cases, 41000 (UR, 8000–75000) stillbirths, and 67000 (UR, 12000–123000) infant deaths (Figure 7).

4. Describe GBS serotypes colonizing mothers and causing maternal and infant GBS disease.

**Figure 8.**
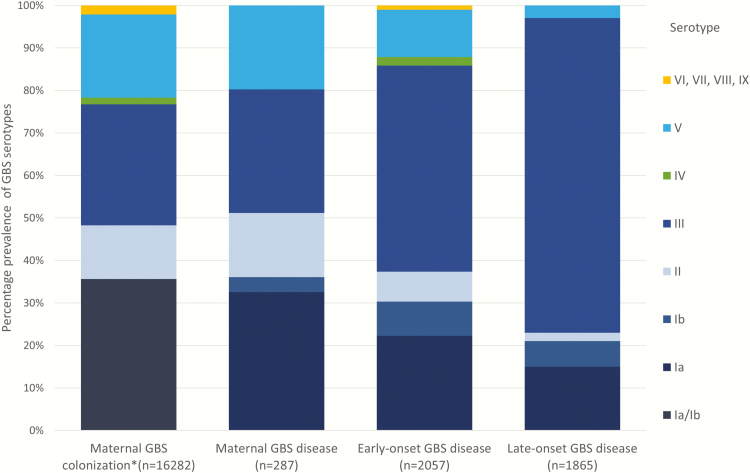
Group B streptococcal (GBS) serotypes colonizing mothers and causing disease in pregnant/postpartum women and infants. *Maternal colonization studies frequently reported Ia/Ib together, so these data are shown pooled. More details are shown in Supplementary Table 5.

Serotype III is the most dominant serotype and colonizes 28% of mothers worldwide. It causes 48% of EOGBS, 74% LOGBS, and 29% of maternal GBS disease (Figure 8). A pentavalent vaccine (Ia/Ib/II/III/V) would cover 96% of worldwide colonizing isolates, 86% of EOGBS disease, 93% of LOGBS disease, and 97% of maternal GBS disease. While there are a limited number of GBS capsular types (n = 10), the distribution by region varies, particularly for maternal GBS colonization; serotypes V, VI, VII, VIII, and IX are more commonly reported in South-Eastern Asia (23%) (Supplementary Table 5).

## DISCUSSION

**Figure 9.**
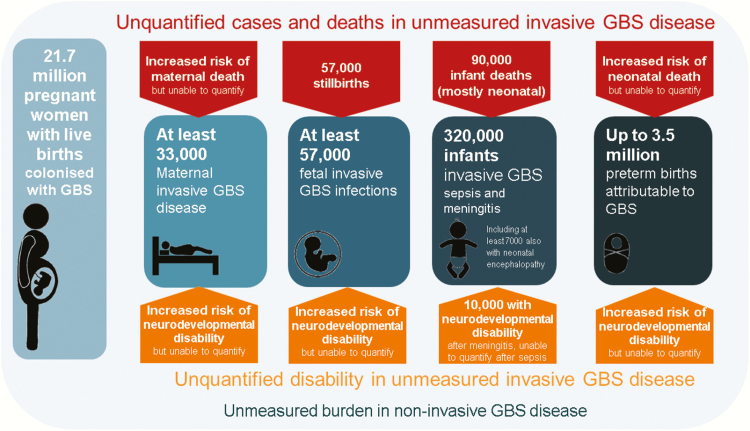
Summary of outcomes and measurement gaps in terms of deaths and disability from group B *Streptococcus* (GBS) in pregnant women, stillbirths, and infants worldwide in 2015. More details of cases and outcomes are shown in Supplementary Figures 4, 6, 7, 10–12.

GBS is established as a leading cause of infant disease, particularly in the first week after birth, as evidenced by our estimation of 205000 (UR, 101000–327000) neonates with EOGBS worldwide. Furthermore, there are a minimum 33000 (UR, 13–52000) maternal GBS cases, 57000 (UR, 12000–104000) fetal infections/stillbirths, and 114000 (UR, 44000–326000) infants with LOGBS. Up to 3.5 million preterm births could be attributable to maternal GBS infection/colonization worldwide (Figure 9).

Importantly, GBS is also a significant cause of death, with 57000 (UR, 12000–104000) stillbirths and 90000 (UR, 36000–169000) infant deaths estimated in 2015. IAP prevented an estimated 3000 (UR, 0–108000) early neonatal deaths in 2015, mainly in high-income contexts. A maternal GBS vaccine, for which candidates are in development (Table 5), with 80% efficacy and 90% coverage could prevent 108000 (UR, 20000–198000) fetal and infant deaths. GBS accounts for more than the total number of deaths from mother-to child transmission of human immunodeficiency virus, and more than the combined neonatal deaths from tetanus, pertussis, and respiratory syncytial virus (Table 6), for which maternal vaccines are already in use, or in advanced development.

**Table 5.** Maternal Group B *Streptococcus* Vaccines in Development With Data in the Public Domain

Vaccine Candidate	Manufacturer	Vaccine Construct	Phase				
			Discovery	Preclinical	Phase 1	Phase 2	Program Status
NA	Pfizer	Multivalent CPS conjugate		X			Clinical program start in 2017 [55]
GBS vaccine	Novartis/GSK	Trivalent CPS (serotypes Ia, IIb, III) conjugated to CRM_197_, unadjuvanted				X	Completed safety and immunogenicity in pregnant women. Study completed [27, 56–61]
NA	GSK	Pentavalent (Ia, Ib, II, III, V) CPS-CRM_197_		X			
NA	GSK	Pilus proteins		X			
NA	Biovac	Polyvalent CPS conjugate	X				Program start in 2017
GBS-NN vaccine/ MVX13211	Minervax	N-domains of Rib and Alpha C surface proteins, unadjuvanted or Alhydrogel-adjuvanted			X		Safety and immunogenicity in nonpregnant women. Study completed [26, 62, 63].

Abbreviations: CPS, capsular polysaccharide; GBS, group B Streptococcus; NA, not available.

**Table 6.** Comparison of Annual Estimates of Infectious Etiologies Causing Stillbirth, Infant Disease, and Death Worldwide, Including Those Where Maternal Vaccination Is Used or Could Be Used to Reduce This Burden

Disease	Stillbirths	No. of Neonatal or Other Relevant Deaths Related to Maternal Infection or Nonimmunity	No. of Neonatal/Infant Cases per Year
Group B *Streptococcus*	57000 (12000–103000)	90 000^a^ (41000–185000)	319 000 (119000–417000)
Respiratory syncytial virus	Not applicable	27300^b^ (20700–36200) [64]	NA
Pertussis	NA	2700^c,d^ [65]	NA
Syphilis	200 000 [7]	62 000^c^ [66]	102 000 [66]
Tetanus	Not applicable	34 000^c^ (18000–84000) [67]	NA
HIV/AIDS	9 000 [7]	86 000^e^ (76000–101000) [67]	NA
Malaria	213 000 [7]	NA	NA

Data are presented as estimate (uncertainty range).

Abbreviations: HIV, human immunodeficiency virus; NA, no relevant estimate available.

^a^Young infants (0–89 days).

^b^Infants (0–6 months).

^c^Neonates (0–27 days).

^d^World Health Organization modeling-based estimates approximately 56 700 pertussis deaths in children <5 years of age in 2015 from which the neonatal component is derived [65]. Other work suggests the burden could be higher, with 160 700 deaths (range, 38 000–670 000 with sensitivity analyses) in children <5 years of age [68].

^e^Children <5 years of age but due to mother-to-child transmission.

The compartmental model approach mitigates some of the very substantial problems with low case ascertainment for invasive infant disease, which can result in huge underestimation, especially in low-income contexts. Our comparatively low estimates using infant incidence data are a result of cases being “missed” through lack of access to healthcare, inadequate clinical assessment and suspicion of infection, lack of diagnostic testing, and lack of appropriate laboratory detection methods such as high-quality blood cultures (Figure 6). Sensitivity of microbiological cultures is further reduced if there has been peripartum antibiotic exposure in the mother or infant. These biases are not included in the uncertainty around estimates from incidence or prevalence data, and thus the uncertainty bounds are likely too narrow. In contrast, the wide uncertainty bounds in the compartmental model, a result of including uncertainty at every step, better reflect the true uncertainty in estimation of these outcomes. In addition, in the compartmental model we addressed other sources of underestimation, by adjusting maternal GBS colonization for sampling site and laboratory methods [16] and only applying CFRs from facility data to births with a skilled birth attendant. Those born at home without access to a skilled birth attendant and who develop EOGBS will have very high, but unobserved, and thus unknown, CFR (Table 7). There are, however, limitations to the compartmental model approach, as described in general elsewhere [15], and in particular where parameters are derived from incidence data, such as the ratio of EOGBS to LOGBS disease. This ratio could be affected through differentially low case ascertainment in EOGBS compared to LOGBS disease or vice versa. EOGBS cases can be reduced with difficulties accessing care after birth and/or a high rapid CFR. LOGBS, which more frequently is meningitis, may be differentially reduced if cerebrospinal fluid sampling is infrequently undertaken, as is often the case in Asia [41].

**Table 7.** Data Inputs Into the Compartmental Model by Step, Considering the Main Biases

Compartmental model	Parameter	Care and Measurement Gap and Resultant Biases				Model Data Input Used
		Lack of Access to Care	Poor Quality of Care and Lack of Clinical Assessment	Failure to Take Appropriate Samples due to Lack of Protocols, Skilled Personnel, or Supplies	Poor Quality of Laboratory Methods to Support Pathogen Detection	
Step 1 Colonization	Infants exposed to maternal GBS at birth [16]	Maternal colonization prevalence measured in facilities: could increase or decrease prevalence dependent on risk factors for maternal GBS colonization		Sample-taking can reduce maternal colonization eg, taking a high vaginal swab. This was adjusted for in prevalence data included.	Culture methods such as broth enrichment increase detection of GBS. Where these were not used, we adjusted the prevalence data included.	Maternal colonization prevalence adjusted for swab sample site and culture methods
Step 2: Cases	IAP policy	IAP only where care accessed	Intrapartum antibiotics could be given inappropriately with overuse or underuse			IAP policy applied nationally with estimated coverage
	Risk of EOGBS	EOGBS underestimated where care access low	EOGBS underestimated if clinical assessment limited	EOGBS underestimated if sampling limited	EOGBS underestimated if laboratory methods insensitive	Risks based on IAP policy in counrtry and estimated coverage
	Ratio of EOGBS to LOGBS	May appear lower with lack of access to care especially at the time of birth	Will likely decrease EOGBS and LOGBS but less change to ratio	Will likely decrease EOGBS and LOGBS but less change to ratio	Will likely decrease EOGBS and LOGBS but less change to ratio	Ratio from regional data due to differences in IAP policies and potential for true differences in EOGBS and LOGBS disease incidence; no adjustments made
	Ratio of meningitis to sepsis cases in EOGBS	Ratio may be higher with lack of access to care, if CFR lower in meningitis	Ratio may be lower with poor quality of care and lack of assessment if meningitis not recognized	Ratio may be lower due to insufficient CSF sampling	Ratio may be higher where blood culture detection more difficult than detection in cerebrospinal fluid	Ratio from worldwide data, may be increased or decreased in either direction; no adjustment made
	Ratio of meningitis to sepsis cases in LOGBS	Ratio may be higher with lack of access to care if CFR lower in meningitis	Ratio may be lower with poor quality of care and lack of assessment if meningitis not recognized	Ratio may be lower due to insufficient CSF sampling	Ratio may be higher where blood culture detection more difficult than detection in CSF	Ratio from worldwide data, may be increased or decreased in either direction; no adjustment made
Step 3: Deaths	Case fatality risk in EOGBS	Reduced with low access to care	Increased with lack of appropriate assessment	Reduced if samples not taken from sickest infants		CFR adjusted to increase where access to care reduced (lack of skilled birth attendant).
	Case fatality risk in LOGBS	Reduced with low access to care but likely less of effect than for EOGBS	Increased with lack of appropriate assessment	Reduced if samples not taken from sickest infants		CFR not adjusted for LOGBS, this likely underestimates death.
Step 4: Disability	NDI risk in infant meningitis (EOGBS and LOGBS)	May decrease NDI if more deaths, but may increase if NDI not detected, eg, through premature death	Underdetection of NDI			NDI incidence at 18 months of age all from developed countries, this likely underestimates cases in the rest of world

Abbreviations: CFR, case fatality risk; CSF, cerebrospinal fluid; EOGBS, early-onset group B Streptococcus; GBS, group B Streptococcus; IAP, intrapartum antibiotic prophylaxis; LOGBS, late-onset group B Streptococcus; NDI, neurodevelopmental impairment.

In high-income contexts, reported incidence data are more reliable. In these countries incidence and trends can be monitored, and surveillance data show that GBS remains one of the most important neonatal and young infant pathogens. The United Kingdom, the Netherlands, and France recently reported increases in incidence of infant disease [42–44]. In the era of *Haemophilus influenzae* type b and pneumococcal conjugate vaccines, GBS is now the leading cause of bacterial meningitis in young children in the United Kingdom and the United States [45, 46]. In low- and middle-income contexts, reported incidence data are more subject to the biases in case ascertainment described. However, for Africa, our estimates of cases of infant disease and fetal infection or stillbirth, are consistent with recent reports of high incidence of GBS disease in facilities from Kenya and South Africa, where assessment and sampling recently have been systematic [47, 48]. For Asia, there is more uncertainty as to the burden of GBS disease. Until recently, the incidence of infant GBS disease was thought to be very low. In addition, there are no data on GBS disease in stillbirth from Asia. In our model, with a very high number of live births in Asia, absolute numbers of infants with EOGBS were high, despite the lower maternal colonization prevalence, suggesting that cases are currently underestimated. For stillbirths, if the prevalence of GBS disease in stillbirths is comparable to Africa, rather than high-income contexts, the total number of stillbirths with GBS disease worldwide would almost double. However, the compartmental model could overestimate invasive infant disease and/or stillbirth if there are biological differences, which it does not account for. There may be differences in virulence of GBS strains circulating in the region. GBS clonal complex 17 (ST17), strongly associated with serotype III, is hypervirulent [48, 49] and less frequently reported in Asia, both for maternal colonization [16] and neonatal disease [19].

Our estimates of maternal GBS disease, stillbirths, and infants with invasive GBS disease presenting with neonatal encephalopathy are all likely to be underestimates as they are all subject to similar challenges for case ascertainment as infant invasive disease, and we were not able to include these in the compartmental model. Invasive GBS disease in newborns presenting with neonatal encephalopathy is further underestimated as the data derive mainly from cooling trials in high-income contexts, with strict case definitions (Figure 2) [22]. In addition, we do not attempt to measure the burden of noninvasive in utero infection, which may sensitize the fetus and increase the risk of neonatal encephalopathy (Figure 9). The challenges of estimating noninvasive disease and the potential size of the unquantified burden are illustrated by the data on the attributable cases of preterm birth [21]. Even a small increase in risk of preterm birth attributable to GBS would account for many preterm births. For other pathogens, such as *Streptococcus pneumoniae*, invasive disease among children accounts for only 10% of all serious disease, with the majority (>80%) of deaths occurring from nonbacteremic pneumonia cases. Robust epidemiological data are critical to strengthen the investment case for a GBS vaccine and to firmly establish the true global burden of disease [50]. Vaccine probe studies during phase 3 maternal vaccine trials, or postlicensure, could also be used to contribute to our understanding of the total disease burden, including noninvasive disease [51].

The current mainstay of prevention against infant GBS disease is IAP, which prevented an estimated 29000 (UR, 0–51000) cases of EOGBS in 2015, mainly in high-income contexts [19]. IAP implementation is more common, and more feasible, in high-income contexts [23], because it requires a continuum of care, including a skilled birth attendant able to administer antibiotics intravenously, and with access to laboratories for a microbiological approach, and/or careful assessment for a clinical risk factor–based approach. There is low implementation, or no IAP national policy where health systems have limited infrastructure. In addition, IAP does not target the 204000 (UR, 69000–481000) cases of maternal, fetal, and late-onset infant invasive infection (7–89 days). It is possible that there is some coincident reduction in disease with IAP, particularly in pregnant women [17], but it is likely administered too late in the context of stillbirth [18]. In addition, IAP does not reduce maternal colonization, so there is no reduction in infant exposure and colonization, and consequent late-onset infant disease [19].

Maternal vaccination has the advantage over IAP in that it could leverage off existing antenatal care platforms, as successfully used in high-burden countries to reduce neonatal tetanus, where high coverage has been achieved [52]. It would also be expected to reduce adverse outcomes for invasive disease in pregnant and postpartum women, fetuses/stillbirths, and infants. A maternal GBS vaccine with 80% efficacy and 50% coverage would prevent 127000 (63000–282000) infant and maternal GBS cases, and 60000 (UR, 22000–110000) stillbirths and infant deaths. If coverage were increased to 90%, 229000 (UR, 114000–507000) infant and maternal GBS cases and 108000 (UR, 20000–198000) fetal and infant deaths could be prevented. Maternal GBS vaccination could also reduce the unquantified burden from noninvasive disease, including, but not limited to, preterm birth. Several GBS vaccine candidates are now in active development [26] and these must be subject to appropriate safety and efficacy tests, but vaccine manufacturers are increasingly committed to investing in a GBS vaccine (Table 5).

There are key public health and economic considerations, to which these estimates contribute. These include (1) the estimated vaccine-preventable mortality burden; (2) the estimated scope, size and cost of a licensure trial; and (3) the cost-effectiveness of a maternal GBS vaccine, to inform policy recommendations, vaccine demand, and financing [51, 53, 54]. Cost-effectiveness models for a maternal GBS vaccine thus far have primarily considered GBS sepsis and meningitis as avertable causes of neonatal mortality [53]. Our estimates of the burden of GBS disease in pregnant and postpartum women and stillbirths, as well as infant disease, suggest they may be additional endpoints worthy of inclusion in a GBS vaccine trial.

GBS is a leading cause of invasive infection in infants, but GBS disease in pregnant and postpartum women and stillbirths is also important worldwide. GBS accounts for a far higher burden of young infant mortality than other infectious diseases for which maternal vaccines are under development or in use, such as respiratory syncytial virus, pertussis, or tetanus (Table 6). Despite GBS accounting for only a small proportion of all stillbirths, the absolute number is equal to a quarter of stillbirths attributed to syphilis, for which there is already a screening program. An effective maternal GBS vaccine offers an all-encompassing approach to reducing GBS disease, and, as vaccination strategies can achieve high coverage in even the most challenging settings, it is likely to be a more equitable intervention than IAP. Maternal GBS vaccination has the potential to reduce this disease burden worldwide, within the next generation and including the poorest families (Table 8).

**Table 8.** Key Findings and Implications

What’s new about this? • These are the first systematic estimates of the worldwide burden of GBS and we include outcomes for pregnant and postpartum women, stillbirth, and infants, with later impairment. For infants this includes invasive disease, overlapping with neonatal encephalopathy, and also noting preterm birth–associated GBS as a pathway resulting in deaths and disability. • Data gaps remain a challenge, but the compartmental model includes more national data and is less susceptible to underestimating the burden through low ascertainment of clinical cases, especially in low-income contexts. We have followed international estimation guidelines and data inputs and code are available online [28, 29].
What are the main findings? • Cases: 319000 (UR, 119000–417000) infant and 33000 (UR, 13000–52000) maternal cases of GBS disease; 7000 (UR, 300–19000) infant cases also had neonatal encephalopathy. Fetal infections would be at least the 57000 (UR, 12000–104000) stillbirths. • Deaths: 57000 (UR, 12000–104000) stillbirths and 90000 (UR, 36000–169000) infant deaths, which is more than the total number of deaths from HIV (mother to child transmission), or more than the combined neonatal deaths from tetanus, pertussis, and RSV (Table 6). • Disability: >10000 (UR, 3000–27000) new cases of neurodevelopmental impairment per year due to infant GBS meningitis. • Other outcomes: Up to 3.5 million cases of preterm birth attributable to GBS.
How can the data be improved? • Geographic: more data are needed worldwide, but especially from Asia. • Outcomes: particular gaps include maternal disease, stillbirth, impairment after infant GBS sepsis, and comorbidity with neonatal encephalopathy. Inclusion of GBS assessments in maternal and neonatal cause-of-death studies should be enhanced. • Economic: cost-effectiveness modeling based on these estimates, and translation to DALYs would be a further step before undertaking economic modeling. • Vaccine trials: standardized definitions of vaccine endpoints also enabling comparison of observational data, and informing program monitoring and evaluation.
What does it mean for policy and programs? • Current provision of IAP prevents an estimated 29000 (UR, 0–51000) cases of EOGBS disease. • Maternal vaccination: With 80% efficacy and 90% coverage could prevent 229000 (UR, 114000–507000) infant and maternal GBS cases, 41000 (UR, 8000–75000) stillbirths, and 67000 (UR, 12000–123000) infant deaths.

Abbreviations: DALY, disability-adjusted life-year; EOGBS, early-onset group B Streptococcus; GBS, group B Streptococcus; HIV, human immunodeficiency virus; IAP, intrapartum antibiotic prophylaxis; RSV, respiratory syncytial virus; UR, uncertainty range.

## Supplementary Data

Supplementary materials are available at *Clinical Infectious Diseases* online. Consisting of data provided by the authors to benefit the reader, the posted materials are not copyedited and are the sole responsibility of the authors, so questions or comments should be addressed to the corresponding author.

## Supplementary Material

Supplement-Material

## Appendix: Multiple Regression Model for Maternal GBS Colonization Prevalence

Purpose: Multiple regression models are commonly used to estimate the burden of diseases, particularly for a single parameter, eg, stillbirth rate or preterm birth rate. In the case of GBS, there are usually multiple outcomes that can be better estimated using a stable compartmental model as discussed elsewhere in this supplement [15]. However, we wanted to assess other approaches including whether a multiple regression model could be used to predict the national prevalence of maternal GBS colonization, using national-level covariates. Maternal GBS colonization prevalence is the parameter with the most data available to which a multiple regression model can be applied.

Inputs: Maternal GBS colonization prevalence data (n = 74) were obtained from published and unpublished literature on GBS worldwide, as described elsewhere in this supplement [16]. Fifteen national-level covariates plausibly associated with GBS colonization prevalence were selected for possible inclusion in the model as predictor variables (full details in Supplementary Table 3):

*log adult female obesity* [34]*, skilled attendant at birth (SBA)* [35]*, log antenatal care (4 visits), mean years female education* [32]*, gross national income (GNI)* [36]*, log neonatal mortality rate (NMR)* [37]*, protected at birth against tetanus (PAB), low birthweight rate (LBW)* [38]*, log general fertility rate (GFR)* [39]*, log GINI coefficient* [36]*, proportion cesarean delivery, syphilis index* [40]*, United Nations region, United Nations subregion, and percentage population urban.*

The association between potential covariates and maternal GBS colonization prevalence was examined using scatterplots (Supplementary Figure 1). Univariable analyses were undertaken to quantify the relationship between GBS maternal colonization prevalence, and continuous variables to assess if these predictors performed better when log transformed. Predictors were then assessed for retention in the model by removing one predictor at a time from the model (starting with the predictor with largest Bayesian information criterion [BIC] on univariate analysis), and dropping the predictor if the model was improved (ie, lower BIC compared to the model containing the predictor).

Model equation and fit: The equation of the final model resulting from the process described above was:

$$Log\;\left(GBS\;prevalence_{ij}\right)\;=\;a\;+\;b\left(LBW_{ij}\right)\;+\;c\left(Log\left(GINI_{ij}\right)\right)\;+\;d\left(GNI_{ij}\right)\;+\;e\left(SBA_{ij}\right)\;+\;f\left(UNregion_{ij}\right)\;+\;u_{j}+\;e_{ij}$$

b(), c(), d(), and e() represent functions each involving 2 parameters, f() indicates a 5 parameter function associated with 5 dummy variables representing different United Nations regions, u_j_ represents country-specific random effects, assumed to be independent normally distributed with constant variance, and e_ij_ represents individual data point-level residuals, assumed to be independent normally distributed with constant variance.

We evaluated the model fit by running a series of diagnostic plots including analyses of regression residuals. The results from the diagnostic plots (Supplementary Figure 2A) and the scatterplot of observed vs predicted data (Supplementary Figure 2B) showed evidence that the model fits the data poorly.

**Conclusions**: This modeling approach was hampered by the limited number of data inputs and particularly by the lack of a strong relationship between available covariates and maternal GBS colonization.

## Notes

***Author contributions.*** The concept of the estimates and the technical oversight of the series were led by J. E. L. and A. C. S. The analyses and first draft of the paper were undertaken by A. C. S. with F. B. and J. E. L. The multiple regression analyses were done by F. B.; H. B. and S. C. advised on analyses and contributed to revisions of drafts. N. R., M. K., C. T., J. H., and M. M. input data for the analyses. The GBS Estimates Expert Advisory Group (C. J. B., L. B., M. G., P. T. H., S. A. M., M. I., K. L. D., C. R., S. K. S., S. J. S., A. S., J. V.) contributed to the conceptual process throughout, notably on the disease schema and data inputs. All the authors reviewed and gave input to the manuscript.

***Acknowledgments.*** We thank Anne C. C. Lee for allowing us to use the individual country estimates of neonatal encephalopathy to apply to 2015 births; Claudia da Silva and Sadie Sareen for assistance organizing Expert Advisory Group meetings; and Alegria Perez for compiling the author signatures.

***Financial support.*** This work was supported by a grant to the London School of Hygiene & Tropical Medicine from the Bill & Melinda Gates Foundation (Grant ID: OPP1131158).

***Supplement sponsorship.*** This article appears as part of the supplement “The Burden of Group B *Streptococcus* Worldwide for Pregnant Women, Stillbirths, and Children,” sponsored by the Bill & Melinda Gates Foundation and coordinated by the London School of Hygiene & Tropical Medicine.

***Potential conflicts of interest.*** Many contributors to this supplement have received funding for their research from foundations, especially the Bill & Melinda Gates Foundation, and several from the Wellcome Trust, the Medical Research Council UK, the Thrasher Foundation, the Meningitis Research Foundation, and one individual from the US National Institutes of Health. Members of the Expert Advisory Group received reimbursement for travel expenses to attend working meetings related to this series. A. S.-t. M. works for the Bill & Melinda Gates Foundation. C. J. B. has served as a member of the Presidential Advisory Committee for Seqirus Inc and of the of the CureVac Inc Scientific Advisory Committee, as well as undertaken consultancy work for Pfizer Inc. C. C. has received institutional compensation from Novartis for conducting GBS studies. P. T. H. has been a consultant to Novartis and Pfizer on GBS vaccines but received no funding for these activities. M. I. has undertaken sponsored research from Pfizer on pneumococcal disease in adults and from Belpharma Eumedica (Belgium) on temocillin antimicrobial susceptibility in Enterobacteriaceae. K. L. D. has received funding by the Bill & Melinda Gates Foundation to work on research on GBS serocorrelates of protection to inform vaccine trials, and travel expenses from Pfizer to attend a meeting on an investigator-led project on GBS. S. A. M. has collaborated on GBS grants funded by GlaxoSmithKline and by Pfizer and received personal fees for being member of its advisory committee; he has also collaborated on a GBS grant funded by Minervax. All other authors report no potential conflicts of interest. All authors have submitted the ICMJE Form for Disclosure of Potential Conflicts of Interest. Conflicts that the editors consider relevant to the content of the manuscript have been disclosed.
