# Supplementary material for: Estimates of the Burden of Group B Streptococcal Disease Worldwide for Pregnant Women, Stillbirths, and Children
Source: Clin Infect Dis. 2017 Nov 6;65(Suppl 2):S200–19. doi: 10.1093/cid/cix664 (PMC5849940; doi:10.1093/cid/cix664)

The burden of Group B *Streptococcus* for pregnant women, stillbirth and children

Paper 11: Estimates of the burden of Group B Streptococcal disease worldwide for pregnant women, stillbirths and children

Supplementary information

Contents

The burden of Group B Streptococcus for pregnant women, stillbirth and children 1

Paper 11: Estimates of the burden of Group B Streptococcal disease worldwide for pregnant women, stillbirths and children 1

Supplementary information 1

Supplementary Table S1: Data inputs to the compartmental model 3

Supplementary Table S2: Data inputs to single cause (incidence or prevalence) models for stillbirth, pregnant women, neonatal encephalopathy 11

Supplementary Table S3: Multiple Regression Modelling for Maternal GBS colonization 18

Supplementary Table S4: Summary table of ranges of risk ratios and estimates of preterm births attributable to GBS. 20

Supplementary Table S5: Summary table of GBS serotypes for maternal colonization, maternal disease and infant disease 21

Supplementary Figure S1: Scatter plots for maternal GBS colonization vs. covariates 23

Supplementary Figure S2A: Diagnostic plots for the GBS maternal colonization prediction regression model 25

Supplementary Figure S2B: Scatterplot of observed vs predicted data for the GBS maternal colonization prediction regression model 26

Supplementary Figure S3: Exposed live born infants to maternal Group B Streptococcus colonization, by sub-region for 2015. 27

Supplementary Figure S4: Early onset and late onset Group B Streptococcal disease cases, by sub-region for 2015. 28

Supplementary Figure S5: Sensitivity analysis: Late onset infant Group B Streptococcal disease using a fixed ratio across regions for EOGBS:LOGBS, by sub-region for 2015. 29

Supplementary Figure S6: Early onset and late onset Group B Streptococcal disease infant deaths, by sub-region for 2015. 30

Supplementary Figure S7: Neurodevelopmental impairment (moderate-severe) after GBS meningitis, by sub-region for 2015 31

Supplementary Figure S8: Comparison of estimates of infants with EOGBS from compartmental model with incidence data, by sub-region for 2015 32

Supplementary Figure S9: Comparison of estimates of infants with LOGBS from compartmental model compared to incidence data, by sub-region for 2015 33

Supplementary Figure S10: Neonatal encephalopathy as a subset of infant invasive GBS disease cases, by sub-region for 2015 34

Supplementary Figure S11 Maternal GBS disease cases (minimum estimates), by sub-region for 2015 35

Supplementary Figure S12: Stillbirths with GBS disease, by sub-region for 2015 36

Supplementary Figure S13: Stillbirths with GBS disease, sensitivity analysis applying estimate from Africa to regions with no data by sub-region for 2015 37

Supplementary Table S1: Data inputs to the compartmental model

Country	Livebirths	Stillbirths	Maternal colonization	IAP risk	EO: LOGBS	Skilled birth	Death no care	Death early	Death late	Early meningitis	Late meningitis	Impairment
**Burundi**	462,790	12,661	0.194	0.011	1.02	0.66	0.9	0.27	0.12	0.12	0.42	0.18
**Comoros**	26,158	824	0.194	0.011	1.02	0.87	0.9	0.27	0.12	0.12	0.42	0.18
**Djibouti**	22,075	791	0.194	0.011	1.02	0.88	0.9	0.27	0.12	0.12	0.42	0.18
**Eritrea**	166,105	3,826	0.194	0.011	1.02	0.41	0.9	0.27	0.12	0.12	0.42	0.18
**Ethiopia**	3,155,752	96,531	0.135	0.011	1.02	0.11	0.9	0.27	0.12	0.12	0.42	0.18
**Kenya**	1,519,845	34,985	0.115	0.011	1.02	0.49	0.9	0.27	0.12	0.12	0.42	0.18
**Madagascar**	821,799	15,235	0.194	0.011	1.02	0.48	0.9	0.27	0.12	0.12	0.42	0.18
**Mozambique**	1,062,694	20,724	0.194	0.011	1.02	0.62	0.9	0.27	0.12	0.12	0.42	0.18
**Mauritius**	14,161	136	0.194	0.00788	1.02	0.76	0.9	0.27	0.12	0.12	0.42	0.18
**Malawi**	629,378	14,036	0.212	0.011	1.02	0.87	0.9	0.27	0.12	0.12	0.42	0.18
**Rwanda**	336,853	5,925	0.194	0.011	1.02	0.74	0.9	0.27	0.12	0.12	0.42	0.18
**Somalia**	445,649	16,400	0.194	0.011	1.02	0.14	0.9	0.27	0.12	0.12	0.42	0.18
**South Sudan**	433,425	13,429	0.194	0.011	1.02	0.17	0.9	0.27	0.12	0.12	0.42	0.18
**Seychelles**	1,668	16	0.194	0.011	1.02	0.99	0.9	0.27	0.12	0.12	0.42	0.18
**Tanzania**	2,051,869	47,060	0.194	0.011	1.02	0.58	0.9	0.27	0.12	0.12	0.42	0.18
**Uganda**	1,590,258	34,151	0.194	0.011	1.02	0.63	0.9	0.27	0.12	0.12	0.42	0.18
**Zambia**	616,264	13,126	0.194	0.011	1.02	0.59	0.9	0.27	0.12	0.12	0.42	0.18
**Zimbabwe**	546,217	11,493	0.194	0.011	1.02	0.70	0.9	0.27	0.12	0.12	0.42	0.18
**Angola**	1,091,655	30,655	0.239	0.011	1.02	0.64	0.9	0.27	0.12	0.12	0.42	0.18
**Central African Republic**	164,159	5,843	0.239	0.011	1.02	0.48	0.9	0.27	0.12	0.12	0.42	0.18
**Cameroon**	819,886	16,365	0.239	0.011	1.02	0.58	0.9	0.27	0.12	0.12	0.42	0.18
**Democratic Republic of the Congo**	3,131,283	87,780	0.239	0.011	1.02	0.86	0.9	0.27	0.12	0.12	0.42	0.18
**Congo**	163,793	2,504	0.239	0.011	1.02	0.94	0.9	0.27	0.12	0.12	0.42	0.18
**Gabon**	51,507	729	0.239	0.011	1.02	0.92	0.9	0.27	0.12	0.12	0.42	0.18
**Equatorial Guinea**	28,525	469	0.239	0.011	1.02	0.77	0.9	0.27	0.12	0.12	0.42	0.18

Country	Livebirths	Stillbirths	Maternal colonization	IAP risk	EO: LOGBS	Skilled birth	Death no care	Death early	Death late	Early meningitis	Late meningitis	Impairment
Sao Tome and Principe	6,347	105	0.239	0.011	1.02	0.89	0.9	0.27	0.12	0.12	0.42	0.18
Chad	605,315	25,170	0.239	0.011	1.02	0.17	0.9	0.27	0.12	0.12	0.42	0.18
Algeria	953,127	18,774	0.229	0.011	1.02	0.98	0.9	0.27	0.12	0.12	0.42	0.18
Egypt	2,798,265	34,656	0.229	0.011	1.02	0.93	0.9	0.27	0.12	0.12	0.42	0.18
Libya	124,224	1,101	0.229	0.011	1.02	1.00	0.9	0.27	0.12	0.12	0.42	0.18
Morocco	721,927	18,144	0.229	0.011	1.02	0.81	0.9	0.27	0.12	0.12	0.42	0.18
Sudan	1,294,181	32,338	0.229	0.011	1.02	0.14	0.9	0.27	0.12	0.12	0.42	0.18
Tunisia	210,239	1,944	0.229	0.011	1.02	0.83	0.9	0.27	0.12	0.12	0.42	0.18
Botswana	57,899	895	0.289	0.011	1.02	1.00	0.9	0.27	0.12	0.12	0.42	0.18
Lesotho	62,526	1,245	0.289	0.011	1.02	0.73	0.9	0.27	0.12	0.12	0.42	0.18
Namibia	74,688	850	0.289	0.011	1.02	0.90	0.9	0.27	0.12	0.12	0.42	0.18
Swaziland	36,578	457	0.289	0.011	1.02	0.86	0.9	0.27	0.12	0.12	0.42	0.18
South Africa	1,012,822	17,985	0.289	0.011	1.02	0.97	0.9	0.27	0.12	0.12	0.42	0.18
Benin	375,949	11,746	0.175	0.011	1.02	0.87	0.9	0.27	0.12	0.12	0.42	0.18
Burkina Faso	687,130	14,911	0.175	0.011	1.02	0.72	0.9	0.27	0.12	0.12	0.42	0.18
Côte d'Ivoire	830,273	22,769	0.175	0.011	1.02	0.67	0.9	0.27	0.12	0.12	0.42	0.18
Cabo Verde	10,880	158	0.175	0.011	1.02	0.94	0.9	0.27	0.12	0.12	0.42	0.18
Ghana	897,610	20,841	0.175	0.011	1.02	0.64	0.9	0.27	0.12	0.12	0.42	0.18
Guinea	456,482	9,856	0.175	0.011	1.02	0.51	0.9	0.27	0.12	0.12	0.42	0.18
Gambia	82,631	2,020	0.175	0.011	1.02	0.63	0.9	0.27	0.12	0.12	0.42	0.18
Guinea-Bissau	65,143	2,482	0.175	0.011	1.02	0.42	0.9	0.27	0.12	0.12	0.42	0.18
Liberia	151,208	3,309	0.175	0.011	1.02	0.65	0.9	0.27	0.12	0.12	0.42	0.18
Mali	725,908	24,410	0.175	0.011	1.02	0.43	0.9	0.27	0.12	0.12	0.42	0.18
Mauritania	131,526	3,659	0.175	0.011	1.02	0.71	0.9	0.27	0.12	0.12	0.42	0.18
Niger	951,289	36,216	0.175	0.011	1.02	0.33	0.9	0.27	0.12	0.12	0.42	0.18
Nigeria	7,000,170	313,706	0.145	0.011	1.02	0.47	0.9	0.27	0.12	0.12	0.42	0.18
Senegal	579,550	14,540	0.175	0.011	1.02	0.59	0.9	0.27	0.12	0.12	0.42	0.18
Sierra Leone	218,202	5,447	0.175	0.011	1.02	0.66	0.9	0.27	0.12	0.12	0.42	0.18
Togo	250,697	8,865	0.175	0.011	1.02	0.55	0.9	0.27	0.12	0.12	0.42	0.18

Country	Livebirths	Stillbirths	Maternal colonization	IAP risk	EO: LOGBS	Skilled birth	Death no care	Death early	Death late	Early meningitis	Late meningitis	Impairment
**Kazakhstan**	392,041	2,556	0.128	0.011	5.60	0.99	0.9	0.14	0.05	0.12	0.42	0.18
**Kyrgyzstan**	172,571	1,783	0.128	0.011	5.60	0.99	0.9	0.14	0.05	0.12	0.42	0.18
**Tajikistan**	267,987	3,799	0.128	0.011	5.60	0.91	0.9	0.14	0.05	0.12	0.42	0.18
**Turkmenistan**	114,191	1,977	0.128	0.011	5.60	0.99	0.9	0.14	0.05	0.12	0.42	0.18
**Uzbekistan**	669,214	8,155	0.128	0.011	5.60	1.00	0.9	0.14	0.05	0.12	0.42	0.18
**China**	17,000,000	122,341	0.113	0.011	5.60	0.99	0.9	0.14	0.05	0.12	0.42	0.18
**Republic of Korea**	458,169	980	0.111	0.00788	5.60	1.00	0.9	0.14	0.05	0.12	0.42	0.18
**Mongolia**	71,083	522	0.111	0.011	5.60	0.99	0.9	0.14	0.05	0.12	0.42	0.18
**Democratic People's Republic of Korea**	377,142	5,161	0.083	0.011	5.60	0.99	0.9	0.14	0.05	0.12	0.42	0.18
**Brunei Darussalam**	7,670	50	0.144	0.011	5.60	1.00	0.9	0.14	0.05	0.12	0.42	0.18
**Indonesia**	5,475,597	73,435	0.144	0.011	5.60	0.84	0.9	0.14	0.05	0.12	0.42	0.18
**Cambodia**	356,899	4,311	0.144	0.011	5.60	0.74	0.9	0.14	0.05	0.12	0.42	0.18
**Lao People's Democratic Republic**	173,409	4,214	0.144	0.011	5.60	0.42	0.9	0.14	0.05	0.12	0.42	0.18
**Myanmar**	920,538	18,745	0.144	0.011	5.60	0.74	0.9	0.14	0.05	0.12	0.42	0.18
**Malaysia**	551,005	3,242	0.144	0.011	5.60	0.99	0.9	0.14	0.05	0.12	0.42	0.18
**Philippines**	2,349,262	25,811	0.144	0.011	5.60	0.76	0.9	0.14	0.05	0.12	0.42	0.18
**Singapore**	53,467	137	0.144	0.0032	5.60	1.00	0.9	0.14	0.05	0.12	0.42	0.18
**Thailand**	740,060	3,697	0.157	0.011	5.60	1.00	0.9	0.14	0.05	0.12	0.42	0.18
**Timor-Leste**	52,089	943	0.144	0.011	5.60	0.33	0.9	0.14	0.05	0.12	0.42	0.18
**Viet Nam**	1,572,561	16,122	0.144	0.011	5.60	0.93	0.9	0.14	0.05	0.12	0.42	0.18
**Afghanistan**	1,020,894	28,056	0.125	0.011	5.60	0.52	0.9	0.14	0.05	0.12	0.42	0.18
**Bangladesh**	3,192,169	83,060	0.112	0.011	5.60	0.41	0.9	0.14	0.05	0.12	0.42	0.18
**Bhutan**	12,368	200	0.125	0.011	5.60	0.89	0.9	0.14	0.05	0.12	0.42	0.18
**India**	25,100,000	592,086	0.096	0.011	5.60	0.57	0.9	0.14	0.05	0.12	0.42	0.18
**Iran (Islamic Republic of)**	1,347,298	8,726	0.157	0.0094	5.60	0.98	0.9	0.14	0.05	0.12	0.42	0.18

Country	Livebirths	Stillbirths	Maternal colonization	IAP risk	EO: LOGBS	Skilled birth	Death no care	Death early	Death late	Early meningitis	Late meningitis	Impairment
**Sri Lanka**	311,658	1,530	0.125	0.011	5.60	0.99	0.9	0.14	0.05	0.12	0.42	0.18
**Maldives**	7,841	61	0.125	0.011	5.60	0.99	0.9	0.14	0.05	0.12	0.42	0.18
**Nepal**	551,895	10,332	0.125	0.011	5.60	0.46	0.9	0.14	0.05	0.12	0.42	0.18
**Pakistan**	5,379,034	242,556	0.125	0.011	5.60	0.58	0.9	0.14	0.05	0.12	0.42	0.18
**United Arab Emirates**	96,732	719	0.158	0.011	5.60	1.00	0.9	0.14	0.05	0.12	0.42	0.18
**Armenia**	40,682	568	0.147	0.011	5.60	1.00	0.9	0.14	0.05	0.12	0.42	0.18
**Azerbaijan**	234,459	3,925	0.147	0.011	5.60	0.99	0.9	0.14	0.05	0.12	0.42	0.18
**Bahrain**	21,713	121	0.147	0.011	5.60	0.99	0.9	0.14	0.05	0.12	0.42	0.18
**Cyprus**	12,930	47	0.147	0.011	5.60	0.94	0.9	0.14	0.05	0.12	0.42	0.18
**Georgia**	56,621	644	0.147	0.011	5.60	1.00	0.9	0.14	0.05	0.12	0.42	0.18
**Iraq**	1,221,858	19,285	0.147	0.011	5.60	0.93	0.9	0.14	0.05	0.12	0.42	0.18
**Israel**	175,315	737	0.121	0.011	5.60	1.00	0.9	0.14	0.05	0.12	0.42	0.18
**Jordan**	206,009	2,183	0.147	0.0094	5.60	1.00	0.9	0.14	0.05	0.12	0.42	0.18
**Kuwait**	69,315	354	0.148	0.011	5.60	0.99	0.9	0.14	0.05	0.12	0.42	0.18
**Lebanon**	102,257	1,020	0.262	0.011	5.60	1.00	0.9	0.14	0.05	0.12	0.42	0.18
**Oman**	81,080	691	0.147	0.0094	5.60	0.99	0.9	0.14	0.05	0.12	0.42	0.18
**Occupied Palestinian Territory**	150,068	1,131	0.147	0.011	5.60	0.79	0.9	0.14	0.05	0.12	0.42	0.18
**Qatar**	28,590	165	0.147	0.011	5.60	1.00	0.9	0.14	0.05	0.12	0.42	0.18
**Saudi Arabia**	615,934	8,702	0.221	0.011	5.60	0.99	0.9	0.14	0.05	0.12	0.42	0.18
**Syrian Arab Republic**	423,732	4,777	0.147	0.011	5.60	0.97	0.9	0.14	0.05	0.12	0.42	0.18
**Turkey**	1,405,609	9,914	0.105	0.011	5.60	0.95	0.9	0.14	0.05	0.12	0.42	0.18
**Yemen**	825,118	24,646	0.147	0.011	5.60	0.40	0.9	0.14	0.05	0.12	0.42	0.18
**Albania**	45,033	180	0.192	0.0032	1.82	1.00	0	0.05	0.04	0.12	0.42	0.18
**Andorra**	763	1	0.192	0.0032	1.82	1.00	0	0.05	0.04	0.12	0.42	0.18
**Australia**	298,489	813	0.238	0.0032	1.82	0.99	0	0.05	0.04	0.12	0.42	0.18
**Austria**	82,503	300	0.146	0.0032	1.82	0.99	0	0.05	0.04	0.12	0.42	0.18
**Belgium**	126,183	382	0.192	0.0032	1.82	1.00	0	0.05	0.04	0.12	0.42	0.18

Country	Livebirths	Stillbirths	Maternal colonization	IAP risk	EO: LOGBS	Skilled birth	Death no care	Death early	Death late	Early meningitis	Late meningitis	Impairment
**Bulgaria**	59,872	345	0.192	0.0032	1.82	1.00	0	0.05	0.04	0.12	0.42	0.18
**Bosnia and Herzegovina**	31,100	170	0.192	0.0032	1.82	1.00	0	0.05	0.04	0.12	0.42	0.18
**Belarus**	125,820	373	0.192	0.0032	1.82	1.00	0	0.05	0.04	0.12	0.42	0.18
**Canada**	369,043	1,162	0.205	0.0032	1.82	0.99	0	0.05	0.04	0.12	0.42	0.18
**Switzerland**	85,621	241	0.187	0.0032	1.82	1.00	0	0.05	0.04	0.12	0.42	0.18
**Czech Republic**	93,398	234	0.192	0.0032	1.82	1.00	0	0.05	0.04	0.12	0.42	0.18
**Germany**	690,048	1,680	0.184	0.0032	1.82	0.99	0	0.05	0.04	0.12	0.42	0.18
**Denmark**	51,742	90	0.192	0.00788	1.82	0.99	0	0.05	0.04	0.12	0.42	0.18
**Spain**	371,160	1,068	0.155	0.0032	1.82	1.00	0	0.05	0.04	0.12	0.42	0.18
**Estonia**	12,157	32	0.192	0.0032	1.82	1.00	0	0.05	0.04	0.12	0.42	0.18
**Finland**	57,753	107	0.192	0.00788	1.82	1.00	0	0.05	0.04	0.12	0.42	0.18
**France**	784,516	3,719	0.156	0.0032	1.82	0.97	0	0.05	0.04	0.12	0.42	0.18
**United Kingdom**	758,286	2,237	0.228	0.00788	1.82	1.00	0	0.05	0.04	0.12	0.42	0.18
**Greece**	105,974	378	0.134	0.0032	1.82	1.00	0	0.05	0.04	0.12	0.42	0.18
**Croatia**	37,620	75	0.192	0.011	1.82	1.00	0	0.05	0.04	0.12	0.42	0.18
**Hungary**	86,895	320	0.192	0.0032	1.82	1.00	0	0.05	0.04	0.12	0.42	0.18
**Ireland**	67,113	183	0.192	0.011	1.82	1.00	0	0.05	0.04	0.12	0.42	0.18
**Iceland**	4,328	5	0.192	0.00788	1.82	1.00	0	0.05	0.04	0.12	0.42	0.18
**Italy**	475,086	1,592	0.232	0.0032	1.82	1.00	0	0.05	0.04	0.12	0.42	0.18
**Japan**	1,045,891	2,172	0.162	0.0032	1.82	1.00	0	0.05	0.04	0.12	0.42	0.18
**Lithuania**	32,474	105	0.192	0.0032	1.82	1.00	0	0.05	0.04	0.12	0.42	0.18
**Luxembourg**	6,686	19	0.192	0.0032	1.82	1.00	0	0.05	0.04	0.12	0.42	0.18
**Latvia**	15,841	57	0.192	0.0032	1.82	0.99	0	0.05	0.04	0.12	0.42	0.18
**Monaco**	569	3	0.192	0.0032	1.82	1.00	0	0.05	0.04	0.12	0.42	0.18
**Republic of Moldova**	43,144	342	0.192	0.0032	1.82	1.00	0	0.05	0.04	0.12	0.42	0.18
**The former Yugoslav Republic of Macedonia**	24,754	192	0.192	0.0032	1.82	1.00	0	0.05	0.04	0.12	0.42	0.18

Country	Livebirths	Stillbirths	Maternal colonization	IAP risk	EO: LOGBS	Skilled birth	Death no care	Death early	Death late	Early meningitis	Late meningitis	Impairment
**Malta**	3,701	13	0.192	0.0032	1.82	1.00	0	0.05	0.04	0.12	0.42	0.18
**Montenegro**	6,874	27	0.192	0.0032	1.82	1.00	0	0.05	0.04	0.12	0.42	0.18
**Netherlands**	179,900	329	0.214	0.00788	1.82	1.00	0	0.05	0.04	0.12	0.42	0.18
**Norway**	60,780	133	0.261	0.00788	1.82	0.99	0	0.05	0.04	0.12	0.42	0.18
**New Zealand**	59,505	134	0.192	0.00788	1.82	0.97	0	0.05	0.04	0.12	0.42	0.18
**Poland**	361,952	851	0.231	0.0032	1.82	1.00	0	0.05	0.04	0.12	0.42	0.18
**Portugal**	83,613	182	0.192	0.0032	1.82	1.00	0	0.05	0.04	0.12	0.42	0.18
**Romania**	166,225	690	0.192	0.0032	1.82	1.00	0	0.05	0.04	0.12	0.42	0.18
**Russian Federation**	1,948,189	8,747	0.192	0.0032	1.82	1.00	0	0.05	0.04	0.12	0.42	0.18
**San Marino**	241	1	0.192	0.0032	1.82	1.00	0	0.05	0.04	0.12	0.42	0.18
**Serbia**	92,627	554	0.192	0.0032	1.82	1.00	0	0.05	0.04	0.12	0.42	0.18
**Slovakia**	54,038	159	0.192	0.0032	1.82	1.00	0	0.05	0.04	0.12	0.42	0.18
**Slovenia**	21,822	63	0.202	0.0032	1.82	1.00	0	0.05	0.04	0.12	0.42	0.18
**Sweden**	113,046	323	0.227	0.0032	1.82	1.00	0	0.05	0.04	0.12	0.42	0.18
**Ukraine**	432,402	3,841	0.192	0.011	1.82	0.99	0	0.05	0.04	0.12	0.42	0.18
**United States of America**	3,805,387	11,261	0.247	0.0032	1.82	0.99	0	0.05	0.04	0.12	0.42	0.18
**Antigua and Barbuda**	1,486	10	0.347	0.011	1.90	0.99	0.9	0.17	0.06	0.12	0.42	0.18
**Bahamas**	5,746	60	0.347	0.011	1.90	0.99	0.9	0.17	0.06	0.12	0.42	0.18
**Barbados**	3,393	29	0.347	0.011	1.90	1.00	0.9	0.17	0.06	0.12	0.42	0.18
**Cuba**	117,263	726	0.347	0.011	1.90	1.00	0.9	0.17	0.06	0.12	0.42	0.18
**Dominica**	1,079	13	0.347	0.011	1.90	0.99	0.9	0.17	0.06	0.12	0.42	0.18
**Dominican Republic**	216,877	2,426	0.347	0.011	1.90	0.98	0.9	0.17	0.06	0.12	0.42	0.18
**Grenada**	1,988	16	0.347	0.0032	1.90	0.99	0.9	0.17	0.06	0.12	0.42	0.18
**Haiti**	257,348	6,578	0.347	0.011	1.90	0.40	0.9	0.17	0.06	0.12	0.42	0.18
**Jamaica**	39,957	766	0.347	0.011	1.90	0.98	0.9	0.17	0.06	0.12	0.42	0.18
**Saint Kitts and Nevis**	929	7	0.347	0.011	1.90	0.99	0.9	0.17	0.06	0.12	0.42	0.18

Country	Livebirths	Stillbirths	Maternal colonization	IAP risk	EO: LOGBS	Skilled birth	Death no care	Death early	Death late	Early meningitis	Late meningitis	Impairment
**Saint Lucia**	2,811	34	0.347	0.011	1.90	1.00	0.9	0.17	0.06	0.12	0.42	0.18
**Trinidad and Tobago**	18,862	212	0.347	0.011	1.90	1.00	0.9	0.17	0.06	0.12	0.42	0.18
**Saint Vincent and the Grenadines**	1,632	18	0.347	0.011	1.90	0.99	0.9	0.17	0.06	0.12	0.42	0.18
**Belize**	8,429	83	0.171	0.0094	1.90	0.97	0.9	0.17	0.06	0.12	0.42	0.18
**Costa Rica**	67,871	409	0.171	0.011	1.90	0.99	0.9	0.17	0.06	0.12	0.42	0.18
**Guatemala**	443,722	5,362	0.171	0.011	1.90	0.62	0.9	0.17	0.06	0.12	0.42	0.18
**Honduras**	162,476	2,074	0.171	0.011	1.90	0.86	0.9	0.17	0.06	0.12	0.42	0.18
**Mexico**	2,370,825	13,093	0.171	0.0032	1.90	0.99	0.9	0.17	0.06	0.12	0.42	0.18
**Nicaragua**	119,382	895	0.171	0.011	1.90	0.89	0.9	0.17	0.06	0.12	0.42	0.18
**Panama**	75,054	464	0.171	0.011	1.90	0.92	0.9	0.17	0.06	0.12	0.42	0.18
**El Salvador**	104,586	1,286	0.171	0.011	1.90	0.98	0.9	0.17	0.06	0.12	0.42	0.18
**Argentina**	761,440	3,499	0.064	0.0032	1.90	1.00	0.9	0.17	0.06	0.12	0.42	0.18
**Bolivia**	246,082	3,209	0.184	0.011	1.90	0.85	0.9	0.17	0.06	0.12	0.42	0.18
**Brazil**	3,213,929	27,808	0.208	0.0032	1.90	0.99	0.9	0.17	0.06	0.12	0.42	0.18
**Chile**	233,554	716	0.141	0.0032	1.90	1.00	0.9	0.17	0.06	0.12	0.42	0.18
**Colombia**	747,774	6,089	0.184	0.011	1.90	0.99	0.9	0.17	0.06	0.12	0.42	0.18
**Ecuador**	326,837	2,531	0.184	0.011	1.90	0.86	0.9	0.17	0.06	0.12	0.42	0.18
**Guyana**	17,128	301	0.184	0.011	1.90	0.93	0.9	0.17	0.06	0.12	0.42	0.18
**Peru**	622,400	5,623	0.184	0.011	1.90	0.92	0.9	0.17	0.06	0.12	0.42	0.18
**Paraguay**	134,968	1,832	0.184	0.011	1.90	0.90	0.9	0.17	0.06	0.12	0.42	0.18
**Suriname**	9,765	193	0.184	0.011	1.90	0.93	0.9	0.17	0.06	0.12	0.42	0.18
**Uruguay**	48,140	318	0.184	0.011	1.90	1.00	0.9	0.17	0.06	0.12	0.42	0.18
**Venezuela (Bolivarian Republic of)**	599,942	4,308	0.184	0.011	1.90	1.00	0.9	0.17	0.06	0.12	0.42	0.18
**Cook Islands**	353	3	0.128	0.011	5.60	0.96	0.9	0.14	0.05	0.12	0.42	0.18
**Fiji**	17,112	206	0.128	0.011	5.60	0.99	0.9	0.14	0.05	0.12	0.42	0.18

Country	Livebirths	Stillbirths	Maternal colonization	IAP risk	EO: LOGBS	Skilled birth	Death no care	Death early	Death late	Early meningitis	Late meningitis	Impairment
**Micronesia (Federated States of)**	2,461	45	0.128	0.011	5.60	0.88	0.9	0.14	0.05	0.12	0.42	0.18
**Kiribati**	2,839	47	0.128	0.011	5.60	0.86	0.9	0.14	0.05	0.12	0.42	0.18
**Marshall Islands**	1,350	21	0.128	0.011	5.60	0.92	0.9	0.14	0.05	0.12	0.42	0.18
**Niue**	29	0	0.128	0.011	5.60	0.96	0.9	0.14	0.05	0.12	0.42	0.18
**Nauru**	148	2	0.128	0.011	5.60	0.99	0.9	0.14	0.05	0.12	0.42	0.18
**Palau**	311	3	0.128	0.011	5.60	1.00	0.9	0.14	0.05	0.12	0.42	0.18
**Papua New Guinea**	209,275	3,383	0.128	0.011	5.60	0.63	0.9	0.14	0.05	0.12	0.42	0.18
**Solomon Islands**	16,671	298	0.128	0.011	5.60	0.80	0.9	0.14	0.05	0.12	0.42	0.18
**Tonga**	2,534	22	0.128	0.011	5.60	0.98	0.9	0.14	0.05	0.12	0.42	0.18
**Tuvalu**	210	3	0.128	0.011	5.60	0.97	0.9	0.14	0.05	0.12	0.42	0.18
**Vanuatu**	6,787	96	0.128	0.011	5.60	0.85	0.9	0.14	0.05	0.12	0.42	0.18
**Samoa**	4,501	50	0.128	0.011	5.60	0.85	0.9	0.14	0.05	0.12	0.42	0.18

Supplementary Table S2: Data inputs to single cause (incidence or prevalence) models for stillbirth, pregnant women, neonatal encephalopathy

Country	Livebirths	Stillbirths	EO neonatal incidence	LO neonatal incidence	Maternal GBS	GBS stillbirth	Neonatal encephal opathy
**Burundi**	462,790	12,661	0.00046	0.00055	0.00023	0.037	0.0058
**Comoros**	26,158	824	0.00046	0.00055	0.00023	0.037	0.0058
**Djibouti**	22,075	791	0.00046	0.00055	0.00023	0.037	0.0058
**Eritrea**	166,105	3,826	0.00046	0.00055	0.00023	0.037	0.0058
**Ethiopia**	3,155,752	96,531	0.00046	0.00055	0.00023	0.037	0.0058
**Kenya**	1,519,845	34,985	0.00046	0.00055	0.00023	0.037	0.0058
**Madagascar**	821,799	15,235	0.00046	0.00055	0.00023	0.037	0.0058
**Mozambique**	1,062,694	20,724	0.00046	0.00055	0.00023	0.037	0.0058
**Mauritius**	14,161	136	0.00046	0.00055	0.00023	0.037	0.0058
**Malawi**	629,378	14,036	0.00046	0.00055	0.00023	0.037	0.0058
**Rwanda**	336,853	5,925	0.00046	0.00055	0.00023	0.037	0.0058
**Somalia**	445,649	16,400	0.00046	0.00055	0.00023	0.037	0.0058
**South Sudan**	433,425	13,429	0.00046	0.00055	0.00023	0.037	0.0058
**Seychelles**	1,668	16	0.00046	0.00055	0.00023	0.037	0.0058
**United Republic of Tanzania**	2,051,869	47,060	0.00046	0.00055	0.00023	0.037	0.0058
**Uganda**	1,590,258	34,151	0.00046	0.00055	0.00023	0.037	0.0058
**Zambia**	616,264	13,126	0.00046	0.00055	0.00023	0.037	0.0058
**Zimbabwe**	546,217	11,493	0.00046	0.00055	0.00023	0.037	0.0058
**Angola**	1,091,655	30,655	0.00071	0.00065	0.00023	0.037	0.0058
**Central African Republic**	164,159	5,843	0.00071	0.00065	0.00023	0.037	0.0058
**Cameroon**	819,886	16,365	0.00071	0.00065	0.00023	0.037	0.0058
**Democratic Republic of the Congo**	3,131,283	87,780	0.00071	0.00065	0.00023	0.037	0.0058
**Congo**	163,793	2,504	0.00071	0.00065	0.00023	0.037	0.0058

**Gabon**	51,507	729	0.00071	0.00065	0.00023	0.037	0.0058
**Equatorial Guinea**	28,525	469	0.00071	0.00065	0.00023	0.037	0.0058
**Sao Tome and Principe**	6,347	105	0.00071	0.00065	0.00023	0.037	0.0058
**Chad**	605,315	25,170	0.00071	0.00065	0.00023	0.037	0.0058
**Algeria**	953,127	18,774	0.00098	0.00065	0.00023	0.037	0.0058
**Egypt**	2,798,265	34,656	0.00098	0.00065	0.00023	0.037	0.0058
**Libya**	124,224	1,101	0.00098	0.00065	0.00023	0.037	0.0058
**Morocco**	721,927	18,144	0.00098	0.00065	0.00023	0.037	0.0058
**Sudan**	1,294,181	32,338	0.00098	0.00065	0.00023	0.037	0.0058
**Tunisia**	210,239	1,944	0.00098	0.00065	0.00023	0.037	0.0058
**Botswana**	57,899	895	0.00106	0.00093	0.00023	0.037	0.0058
**Lesotho**	62,526	1,245	0.00106	0.00093	0.00023	0.037	0.0058
**Namibia**	74,688	850	0.00106	0.00093	0.00023	0.037	0.0058
**Swaziland**	36,578	457	0.00106	0.00093	0.00023	0.037	0.0058
**South Africa**	1,012,822	17,985	0.00106	0.00093	0.00023	0.037	0.0058
**Benin**	375,949	11,746	0.00025	0.00065	0.00023	0.037	0.0058
**Burkina Faso**	687,130	14,911	0.00025	0.00065	0.00023	0.037	0.0058
**Côte d'Ivoire**	830,273	22,769	0.00025	0.00065	0.00023	0.037	0.0058
**Cabo Verde**	10,880	158	0.00025	0.00065	0.00023	0.037	0.0058
**Ghana**	897,610	20,841	0.00025	0.00065	0.00023	0.037	0.0058
**Guinea**	456,482	9,856	0.00025	0.00065	0.00023	0.037	0.0058
**Gambia**	82,631	2,020	0.00025	0.00065	0.00023	0.037	0.0058
**Guinea-Bissau**	65,143	2,482	0.00025	0.00065	0.00023	0.037	0.0058
**Liberia**	151,208	3,309	0.00025	0.00065	0.00023	0.037	0.0058
**Mali**	725,908	24,410	0.00025	0.00065	0.00023	0.037	0.0058
**Mauritania**	131,526	3,659	0.00025	0.00065	0.00023	0.037	0.0058
**Niger**	951,289	36,216	0.00025	0.00065	0.00023	0.037	0.0058
**Nigeria**	7,000,170	313,706	0.00025	0.00065	0.00023	0.037	0.0058
**Senegal**	579,550	14,540	0.00025	0.00065	0.00023	0.037	0.0058
**Sierra Leone**	218,202	5,447	0.00025	0.00065	0.00023	0.037	0.0058

**Togo**	250,697	8,865	0.00025	0.00065	0.00023	0.037	0.0058
**Kazakhstan**	392,041	2,556	0.00032	0.00004	0.00023	0.01	0.0058
**Kyrgyzstan**	172,571	1,783	0.00032	0.00004	0.00023	0.01	0.0058
**Tajikistan**	267,987	3,799	0.00032	0.00004	0.00023	0.01	0.0058
**Turkmenistan**	114,191	1,977	0.00032	0.00004	0.00023	0.01	0.0058
**Uzbekistan**	669,214	8,155	0.00032	0.00004	0.00023	0.01	0.0058
**China**	17,000,000	122,341	0.00025	0.00038	0.00023	0.01	0.0058
**Republic of Korea**	458,169	980	0.00025	0.00038	0.00023	0.01	0.0058
**Mongolia**	71,083	522	0.00025	0.00038	0.00023	0.01	0.0058
**Democratic People's Republic of Korea**	377,142	5,161	0.00025	0.00038	0.00023	0.01	0.0058
**Brunei Darussalam**	7,670	50	0.00024	0.00003	0.00023	0.01	0.0058
**Indonesia**	5,475,597	73,435	0.00024	0.00003	0.00023	0.01	0.0058
**Cambodia**	356,899	4,311	0.00024	0.00003	0.00023	0.01	0.0058
**Lao People's Democratic Republic**	173,409	4,214	0.00024	0.00003	0.00023	0.01	0.0058
**Myanmar**	920,538	18,745	0.00024	0.00003	0.00023	0.01	0.0058
**Malaysia**	551,005	3,242	0.00024	0.00003	0.00023	0.01	0.0058
**Philippines**	2,349,262	25,811	0.00024	0.00003	0.00023	0.01	0.0058
**Singapore**	53,467	137	0.00024	0.00003	0.00023	0.01	0.0058
**Thailand**	740,060	3,697	0.00024	0.00003	0.00023	0.01	0.0058
**Timor-Leste**	52,089	943	0.00024	0.00003	0.00023	0.01	0.0058
**Viet Nam**	1,572,561	16,122	0.00024	0.00003	0.00023	0.01	0.0058
**Afghanistan**	1,020,894	28,056	0.0002	0.00003	0.00023	0.01	0.0058
**Bangladesh**	3,192,169	83,060	0.0002	0.00003	0.00023	0.01	0.0058
**Bhutan**	12,368	200	0.0002	0.00003	0.00023	0.01	0.0058
**India**	25,100,000	592,086	0.0002	0.00003	0.00023	0.01	0.0058
**Iran (Islamic Republic of)**	1,347,298	8,726	0.0002	0.00003	0.00023	0.01	0.0058
**Sri Lanka**	311,658	1,530	0.0002	0.00003	0.00023	0.01	0.0058
**Maldives**	7,841	61	0.0002	0.00003	0.00023	0.01	0.0058

**Nepal**	551,895	10,332	0.0002	0.00003	0.00023	0.01	0.0058
**Pakistan**	5,379,034	242,556	0.0002	0.00003	0.00023	0.01	0.0058
**United Arab Emirates**	96,732	719	0.00058	0.00004	0.00023	0.01	0.0058
**Armenia**	40,682	568	0.00058	0.00004	0.00023	0.01	0.0058
**Azerbaijan**	234,459	3,925	0.00058	0.00004	0.00023	0.01	0.0058
**Bahrain**	21,713	121	0.00058	0.00004	0.00023	0.01	0.0058
**Cyprus**	12,930	47	0.00058	0.00004	0.00023	0.01	0.0058
**Georgia**	56,621	644	0.00058	0.00004	0.00023	0.01	0.0058
**Iraq**	1,221,858	19,285	0.00058	0.00004	0.00023	0.01	0.0058
**Israel**	175,315	737	0.00058	0.00004	0.00023	0.01	0.0058
**Jordan**	206,009	2,183	0.00058	0.00004	0.00023	0.01	0.0058
**Kuwait**	69,315	354	0.00058	0.00004	0.00023	0.01	0.0058
**Lebanon**	102,257	1,020	0.00058	0.00004	0.00023	0.01	0.0058
**Oman**	81,080	691	0.00058	0.00004	0.00023	0.01	0.0058
**Occupied Palestinian Territory**	150,068	1,131	0.00058	0.00004	0.00023	0.01	0.0058
**Qatar**	28,590	165	0.00058	0.00004	0.00023	0.01	0.0058
**Saudi Arabia**	615,934	8,702	0.00058	0.00004	0.00023	0.01	0.0058
**Syrian Arab Republic**	423,732	4,777	0.00058	0.00004	0.00023	0.01	0.0058
**Turkey**	1,405,609	9,914	0.00058	0.00004	0.00023	0.01	0.0058
**Yemen**	825,118	24,646	0.00058	0.00004	0.00023	0.01	0.0058
**Albania**	45,033	180	0.00037	0.00018	0.00023	0.01	0.0051
**Andorra**	763	1	0.00037	0.00018	0.00023	0.01	0.0051
**Australia**	298,489	813	0.00037	0.00018	0.00023	0.01	0.0051
**Austria**	82,503	300	0.00037	0.00018	0.00023	0.01	0.0051
**Belgium**	126,183	382	0.00037	0.00018	0.00023	0.01	0.0051
**Bulgaria**	59,872	345	0.00037	0.00018	0.00023	0.01	0.0051
**Bosnia and Herzegovina**	31,100	170	0.00037	0.00018	0.00023	0.01	0.0051
**Belarus**	125,820	373	0.00037	0.00018	0.00023	0.01	0.0051
**Canada**	369,043	1,162	0.00037	0.00018	0.00023	0.01	0.0051

**Switzerland**	85,621	241	0.00037	0.00018	0.00023	0.01	0.0051
**Czech Republic**	93,398	234	0.00037	0.00018	0.00023	0.01	0.0051
**Germany**	690,048	1,680	0.00037	0.00018	0.00023	0.01	0.0051
**Denmark**	51,742	90	0.00037	0.00018	0.00023	0.01	0.0051
**Spain**	371,160	1,068	0.00037	0.00018	0.00023	0.01	0.0051
**Estonia**	12,157	32	0.00037	0.00018	0.00023	0.01	0.0051
**Finland**	57,753	107	0.00037	0.00018	0.00023	0.01	0.0051
**France**	784,516	3,719	0.00037	0.00018	0.00023	0.01	0.0051
**United Kingdom**	758,286	2,237	0.00037	0.00018	0.00023	0.01	0.0051
**Greece**	105,974	378	0.00037	0.00018	0.00023	0.01	0.0051
**Croatia**	37,620	75	0.00037	0.00018	0.00023	0.01	0.0051
**Hungary**	86,895	320	0.00037	0.00018	0.00023	0.01	0.0051
**Ireland**	67,113	183	0.00037	0.00018	0.00023	0.01	0.0051
**Iceland**	4,328	5	0.00037	0.00018	0.00023	0.01	0.0051
**Italy**	475,086	1,592	0.00037	0.00018	0.00023	0.01	0.0051
**Japan**	1,045,891	2,172	0.00037	0.00018	0.00023	0.01	0.0051
**Lithuania**	32,474	105	0.00037	0.00018	0.00023	0.01	0.0051
**Luxembourg**	6,686	19	0.00037	0.00018	0.00023	0.01	0.0051
**Latvia**	15,841	57	0.00037	0.00018	0.00023	0.01	0.0051
**Monaco**	569	3	0.00037	0.00018	0.00023	0.01	0.0051
**Republic of Moldova**	43,144	342	0.00037	0.00018	0.00023	0.01	0.0051
**The former Yugoslav Republic of Macedonia**	24,754	192	0.00037	0.00018	0.00023	0.01	0.0051
**Malta**	3,701	13	0.00037	0.00018	0.00023	0.01	0.0051
**Montenegro**	6,874	27	0.00037	0.00018	0.00023	0.01	0.0051
**Netherlands**	179,900	329	0.00037	0.00018	0.00023	0.01	0.0051
**Norway**	60,780	133	0.00037	0.00018	0.00023	0.01	0.0051
**New Zealand**	59,505	134	0.00037	0.00018	0.00023	0.01	0.0051
**Poland**	361,952	851	0.00037	0.00018	0.00023	0.01	0.0051
**Portugal**	83,613	182	0.00037	0.00018	0.00023	0.01	0.0051

**Romania**	166,225	690	0.00037	0.00018	0.00023	0.01	0.0051
**Russian Federation**	1,948,189	8,747	0.00037	0.00018	0.00023	0.01	0.0051
**San Marino**	241	1	0.00037	0.00018	0.00023	0.01	0.0051
**Serbia**	92,627	554	0.00037	0.00018	0.00023	0.01	0.0051
**Slovakia**	54,038	159	0.00037	0.00018	0.00023	0.01	0.0051
**Slovenia**	21,822	63	0.00037	0.00018	0.00023	0.01	0.0051
**Sweden**	113,046	323	0.00037	0.00018	0.00023	0.01	0.0051
**Ukraine**	432,402	3,841	0.00037	0.00018	0.00023	0.01	0.0051
**United States of America**	3,805,387	11,261	0.00037	0.00018	0.00023	0.01	0.0051
**Antigua and Barbuda**	1,486	10	0.00147	0.00019	0.00023	0.01	0.0051
**Bahamas**	5,746	60	0.00147	0.00019	0.00023	0.01	0.0051
**Barbados**	3,393	29	0.00147	0.00019	0.00023	0.01	0.0051
**Cuba**	117,263	726	0.00147	0.00019	0.00023	0.01	0.0051
**Dominica**	1,079	13	0.00147	0.00019	0.00023	0.01	0.0051
**Dominican Republic**	216,877	2,426	0.00147	0.00019	0.00023	0.01	0.0051
**Grenada**	1,988	16	0.00147	0.00019	0.00023	0.01	0.0051
**Haiti**	257,348	6,578	0.00147	0.00019	0.00023	0.01	0.0051
**Jamaica**	39,957	766	0.00147	0.00019	0.00023	0.01	0.0058
**Saint Kitts and Nevis**	929	7	0.00147	0.00019	0.00023	0.01	0.0058
**Saint Lucia**	2,811	34	0.00147	0.00019	0.00023	0.01	0.0058
**Trinidad and Tobago**	18,862	212	0.00147	0.00019	0.00023	0.01	0.0058
**Saint Vincent and the Grenadines**	1,632	18	0.00147	0.00019	0.00023	0.01	0.0058
**Belize**	8,429	83	0.00077	0.00058	0.00023	0.01	0.0058
**Costa Rica**	67,871	409	0.00077	0.00058	0.00023	0.01	0.0058
**Guatemala**	443,722	5,362	0.00077	0.00058	0.00023	0.01	0.0058
**Honduras**	162,476	2,074	0.00077	0.00058	0.00023	0.01	0.0058
**Mexico**	2,370,825	13,093	0.00077	0.00058	0.00023	0.01	0.0058
**Nicaragua**	119,382	895	0.00077	0.00058	0.00023	0.01	0.0058
**Panama**	75,054	464	0.00077	0.00058	0.00023	0.01	0.0058

**El Salvador**	104,586	1,286	0.00077	0.00058	0.00023	0.01	0.0058
**Argentina**	761,440	3,499	0.00034	0.00022	0.00023	0.01	0.0058
**Bolivia**	246,082	3,209	0.00034	0.00022	0.00023	0.01	0.0058
**Brazil**	3,213,929	27,808	0.00034	0.00022	0.00023	0.01	0.0058
**Chile**	233,554	716	0.00034	0.00022	0.00023	0.01	0.0058
**Colombia**	747,774	6,089	0.00034	0.00022	0.00023	0.01	0.0058
**Ecuador**	326,837	2,531	0.00034	0.00022	0.00023	0.01	0.0058
**Guyana**	17,128	301	0.00034	0.00022	0.00023	0.01	0.0058
**Peru**	622,400	5,623	0.00034	0.00022	0.00023	0.01	0.0058
**Paraguay**	134,968	1,832	0.00034	0.00022	0.00023	0.01	0.0058
**Suriname**	9,765	193	0.00034	0.00022	0.00023	0.01	0.0058
**Uruguay**	48,140	318	0.00034	0.00022	0.00023	0.01	0.0058
**Venezuela (Bolivarian Republic of)**	599,942	4,308	0.00034	0.00022	0.00023	0.01	0.0058
**Cook Islands**	353	3	0.00032	0.00004	0.00023	0.01	0.0058
**Fiji**	17,112	206	0.00032	0.00004	0.00023	0.01	0.0058
**Micronesia (Federated States of)**	2,461	45	0.00032	0.00004	0.00023	0.01	0.0058
**Kiribati**	2,839	47	0.00032	0.00004	0.00023	0.01	0.0058
**Marshall Islands**	1,350	21	0.00032	0.00004	0.00023	0.01	0.0058
**Niue**	29	0	0.00032	0.00004	0.00023	0.01	0.0058
**Nauru**	148	2	0.00032	0.00004	0.00023	0.01	0.0058
**Palau**	311	3	0.00032	0.00004	0.00023	0.01	0.0058
**Papua New Guinea**	209,275	3,383	0.00032	0.00004	0.00023	0.01	0.0058
**Solomon Islands**	16,671	298	0.00032	0.00004	0.00023	0.01	0.0058
**Tonga**	2,534	22	0.00032	0.00004	0.00023	0.01	0.0058
**Tuvalu**	210	3	0.00032	0.00004	0.00023	0.01	0.0058
**Vanuatu**	6,787	96	0.00032	0.00004	0.00023	0.01	0.0058
**Samoa**	4,501	50	0.00032	0.00004	0.00023	0.01	0.0058

Supplementary Table S3: Multiple Regression Modelling for Maternal GBS colonization

Data inputs: 74

Covariates: Adult female obesity, skilled attendant at birth (SBA), antenatal care (4 visits), mean years female education, Gross national income (GNI), neonatal mortality rate (NMR), protected at birth against tetanus (PAB), low birthweight rate (LBW), general fertility rate (GFR), GINI coefficient, percentage population urban, proportion c-section, Syphilis index, UN regions, UN subregions **Total:** 15

Univariate analyses: selected lowest BIC between variable and log-transformed value

Covariate	Coefficient	p-value	BIC
**log_obesity**	0.1753	0.011	86.59
**SBA**	0.0057	0.017	87.30
**log_ANC4**	0.3142	0.043	88.81
**Education**	0.0271	0.088	89.93
**GNI**	0.0000035	0.155	90.79
**log_GINI**	-0.3097	0.167	90.89
**Urban**	0.0026	0.205	91.19
**log_NMR**	-0.0532	0.236	91.39
**LBW**	-0.0084	0.362	91.96
**log_GFR**	-0.048	0.651	92.58
**PAB**	0.0026	0.669	92.60

Model fitting process: covariates tried in order of BIC

Covariates tried dropping	BIC	Dropped or retained
**Full**	175.40	
**PAB**	172.66	Dropped from model
**log_GFR**	168.87	Dropped from model
**LBW**	168.27	Retained in model
**log_NMR**	164.61	Dropped from model
**Urban**	160.36	Dropped from model
**log_GINI**	169.66	Retained in model
**GNI**	160.09	Retained in model
**Education**	156.43	Dropped from model
**log_ANC4**	153.08	Dropped from model
**SBA**	154.49	Retained in model
**log_obese**	150.42	Dropped from model
**Subregion**	114.20	Dropped from model
**Region**	116.97	Retained in model
**Syphilis**	106.68	Dropped from model
**C-section**	97.22	Dropped from model

Final equation: $\log(\text{GBS prevalence}_{ij}) = a + b(\text{LBW}_{ij}) + c(\log(\text{GINI}_{ij})) + d(\text{GNI}_{ij}) + e(\text{SBA}_{ij}) + f(\text{region}_{ij}) + u_j + e_{ij}$

Output of the final model:

Variable	Coefficient	p-value	95% CI
**SBA**	0.0140	0.000	.0071305-.020894
**GNI**	-1.31 e-06	0.711	-8.26e-06 5.64e-06
**GINI**	-0.8955	0.007	-1.543414 -.247558
**LBW**	0.0195	0.083	-.0025691 .0416023
**UN region**			
**Africa**	0.5523	0.001	.2144634 .8901618
**America**	0.3228	0.046	.0051606 .6404568
**Asia**	-0.0928	0.454	-.3355977 .1500058
**Oceania**	0.2223	0.381	-.2752256 .7199669

-

Supplementary Table S4: Summary table of ranges of risk ratios and estimates of preterm births attributable to GBS.

Risk ratio	Preterm births attributable to GBS
**1.0**	0
**1.2**	500,000
**1.4**	1,000,000
**1.6**	1,400,000
**1.8**	1,800,000
**2.0**	2,200,000
**2.2**	2,500,000
**2.4**	2,900,000
**2.6**	3,200,000
**2.8**	3,500,000

Supplementary Table S5: Summary table of GBS serotypes for maternal colonization, maternal disease and infant disease

		Maternal GBS colonization								Maternal GBS disease								
		No. countries	No. input	Serotype prevalence %						No. countries	No. input	Serotype prevalence %						
**Regions**	Subregions			Ia/Ib	II	III	IV	V	VI, VII, VIII, IX)			Ia	Ib	II	III	IV	V	VI, VII, VIII, IX
**Developed**		21	7344	33	12	30	3	19	3	5	287	33	4	15	29	0	20	0
**Latin America and the Caribbean**	South America	2	241	55	15	17	0	11	1	-	-	-	-	-	-	-	-	-
	Caribbean	-	-	-	-	-	-	-	-	-	-	-	-	-	-	-	-	-
	Central America	1	338	68	22	11	0	0	0	-	-	-	-	-	-	-	-	-
**Asia**	Eastern Asia	3	2937	32	8	31	0	18	12	-	-	-	-	-	-	-	-	-
	Southern Asia	3	553	36	16	27	0	15	6	-	-	-	-	-	-	-	-	-
	Central Asia	-	-	-	-	-	-	-	-	-	-	-	-	-	-	-	-	-
	South-eastern Asia	4	365	21	16	14	3	23	23	-	-	-	-	-	-	-	-	-
	Western Asia	5	958	28	16	26	4	22	4	-	-	-	-	-	-	-	-	-
**Africa**	Northern Africa	2	58	26	25	25	0	24	0	-	-	-	-	-	-	-	-	-
	Eastern Africa	4	1551	26	6	37	2	28	0	-	-	-	-	-	-	-	-	-
	Middle Africa	2	197	36	14	22	0	28	0	-	-	-	-	-	-	-	-	-
	Western Africa	3	433	26	15	17	0	42	0	-	-	-	-	-	-	-	-	-
	Southern Africa	1	2029	45	9	31	3	13	0	-	-	-	-	-	-	-	-	-
**Oceania**	Oceania	-	-	-	-	-	-	-	-	-	-	-	-	-	-	-	-	-

		Early-onset GBS disease									Late-onset GBS disease								
		No. countries	No. input	Serotype prevalence %							No. countries	No. input	Serotype prevalence %						
**Regions**	Subregions			Ia	Ib	II	III	IV	V	VI, VII, VIII, IX			Ia	Ib	II	III	IV	V	VI, VII, VIII, IX
**Developed**		20	1831	22	8	8	48	2	11	1	18	1658	16	6	3	72	0	3	0
**Latin America and the Caribbean**	South America	1	6	33	-	-	33	-	33	-	-	-	-	-	-	-	-	-	-
	Caribbean	-	-	-	-	-	-	-	-	-	-	-	-	-	-	-	-	-	-
	Central America	1	12	50	-	8	42	-	-	-	1	9	-	-	-	100	-	-	-
**Asia**	Eastern Asia	3	52	16	20	-	41	-	23	-	3	71	7	12	0	56	-	23	1
	Southern Asia	-	-	-	-	-	-	-	-	-	-	-	-	-	-	-	-	-	-
	Central Asia	-	-	-	-	-	-	-	-	-	-	-	-	-	-	-	-	-	-
	South-eastern Asia	-	-	-	-	-	-	-	-	-	-	-	-	-	-	-	-	-	-
	Western Asia	-	-	-	-	-	-	-	-	-	-	-	-	-	-	-	-	-	-
**Africa**	Northern Africa	-	-	-	-	-	-	-	-	-	-	-	-	-	-	-	-	-	-
	Eastern Africa	1	31	23	10	3	61	3	-	-	1	39	8	3	-	89	-	-	-
	Middle Africa	-	-	-	-	-	-	-	-	-	-	-	-	-	-	-	-	-	-
	Western Africa	-	-	-	-	-	-	-	-	-	-	-	-	-	-	-	-	-	-
	Southern Africa	1	125	22	6	6	52	4	10	-	1	88	15	1	-	83	-	1	-
**Oceania**	Oceania	-	-	-	-	-	-	-	-	-	-	-	-	-	-	-	-	-	-

Supplementary Figure S1: Scatter plots for maternal GBS colonization vs. covariates

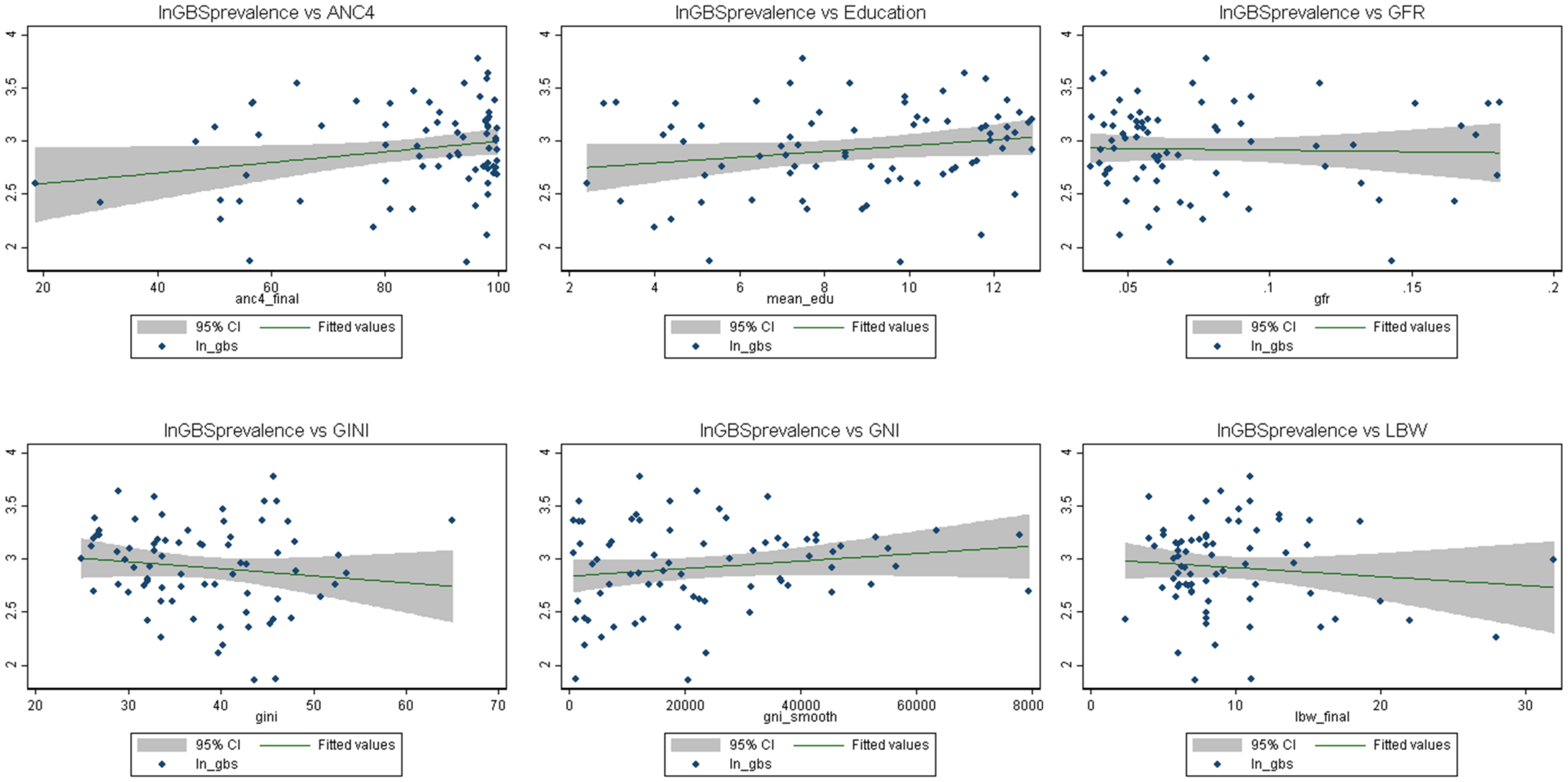

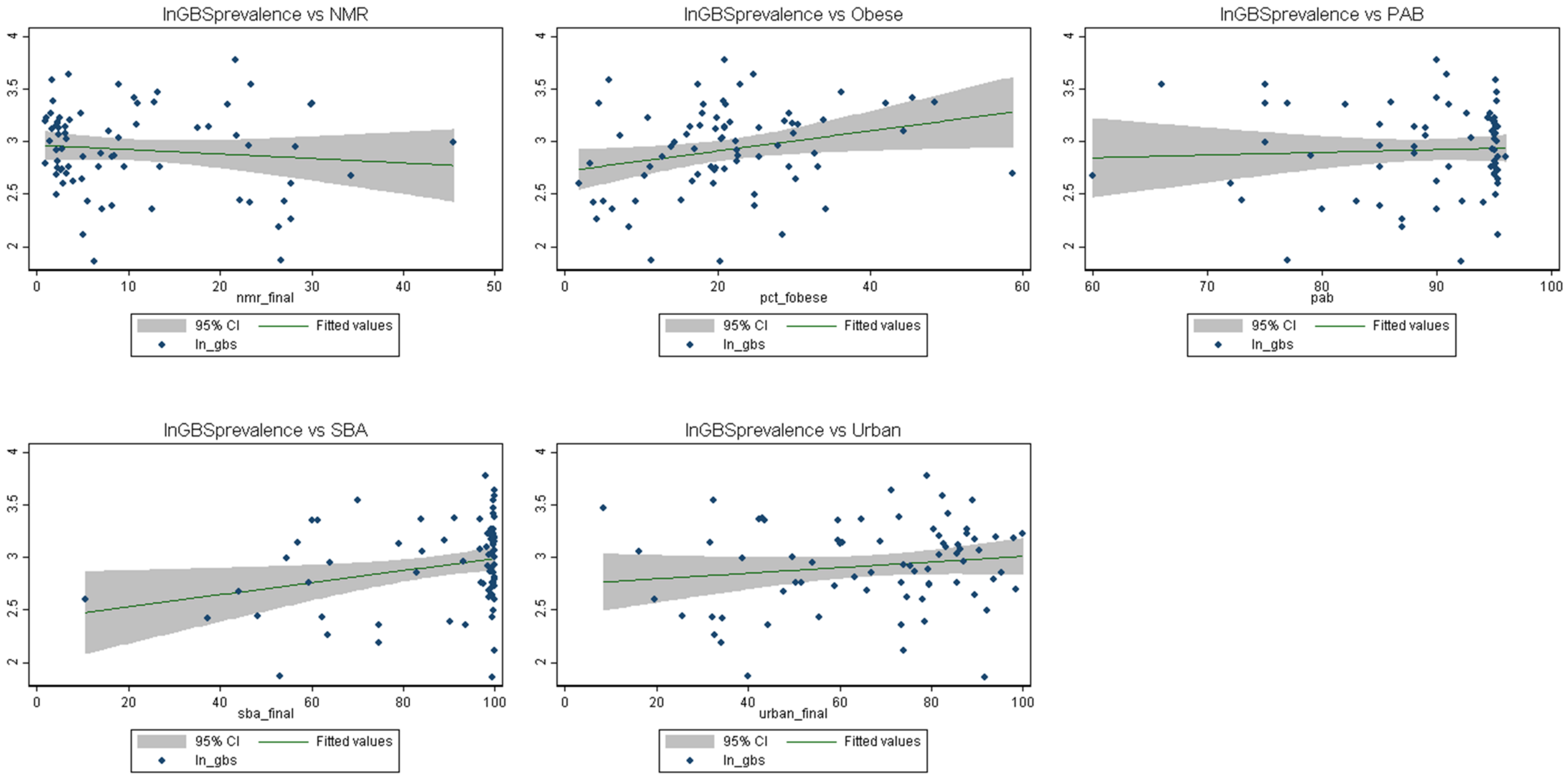

lnGBSprevalence vs NMR
nmr_final
lnGBSprevalence vs Obese
pct_fobese
lnGBSprevalence vs PAB
pab
lnGBSprevalence vs SBA
sba_final
lnGBSprevalence vs Urban
urban_final
95% CI
Fitted values
ln_gbs

Supplementary Figure S2A: Diagnostic plots for the GBS maternal colonization prediction regression model

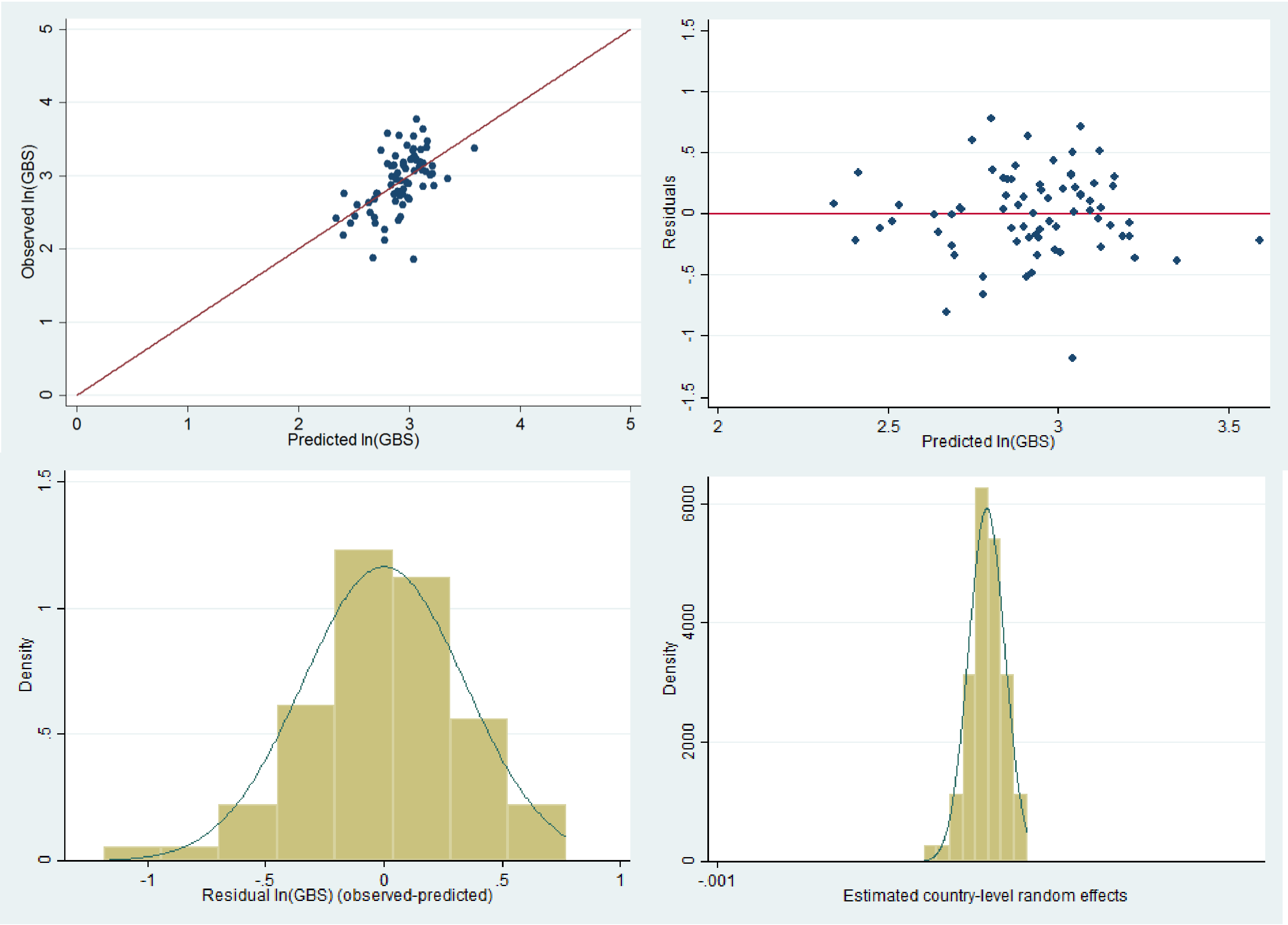

Supplementary Figure S2B: Scatterplot of observed vs predicted data for the GBS maternal colonization prediction regression model

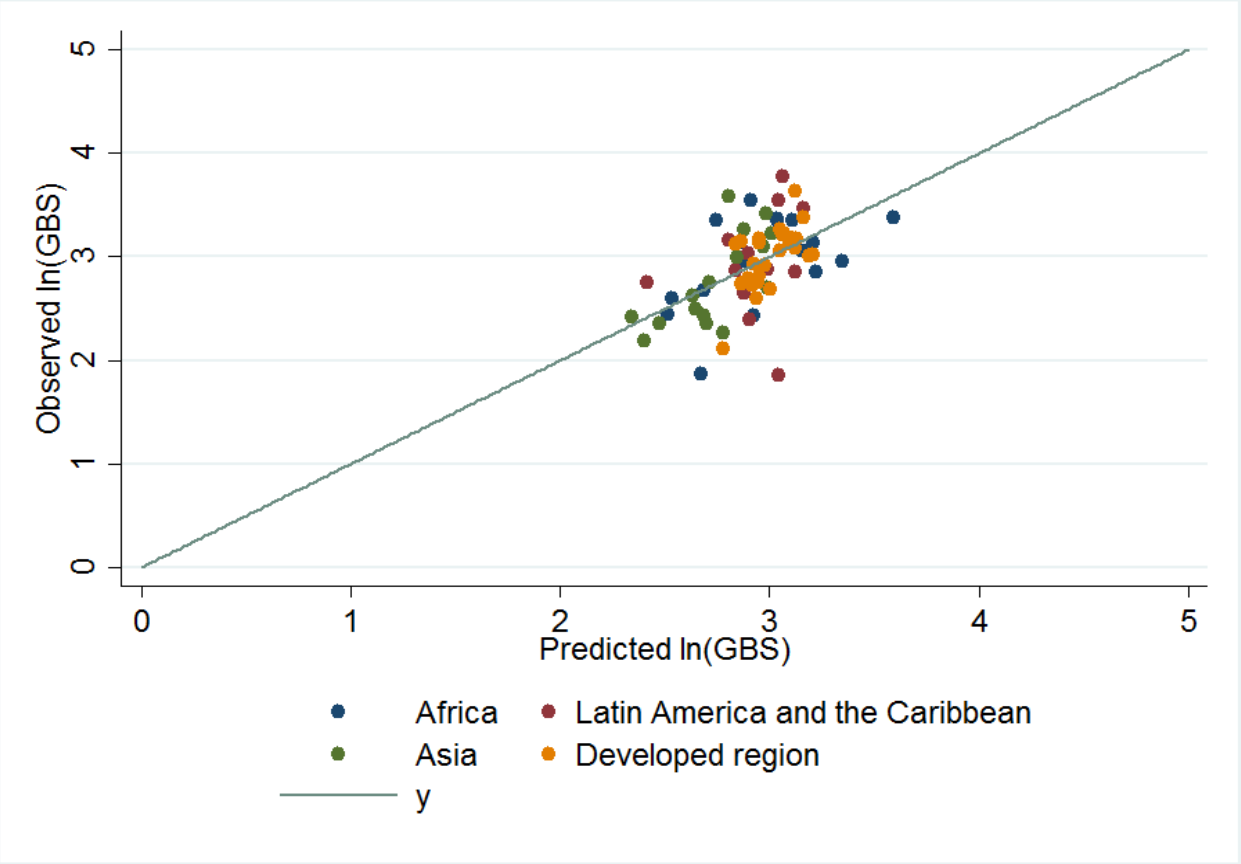

Supplementary Figure S3: Exposed live born infants to maternal Group B Streptococcus colonization, by sub-region for 2015.

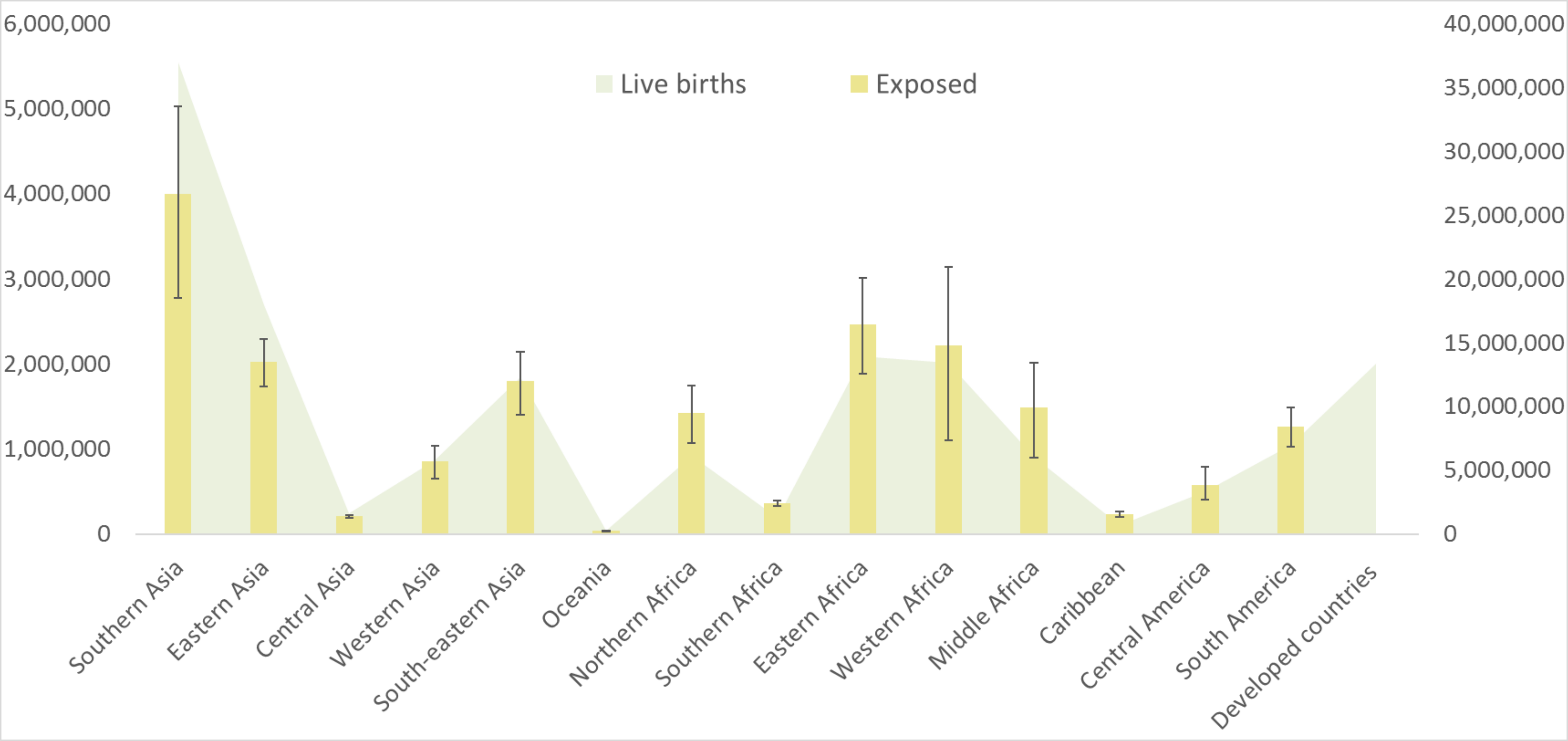

Supplementary Figure S4: Early onset and late onset Group B Streptococcal disease cases, by sub-region for 2015.

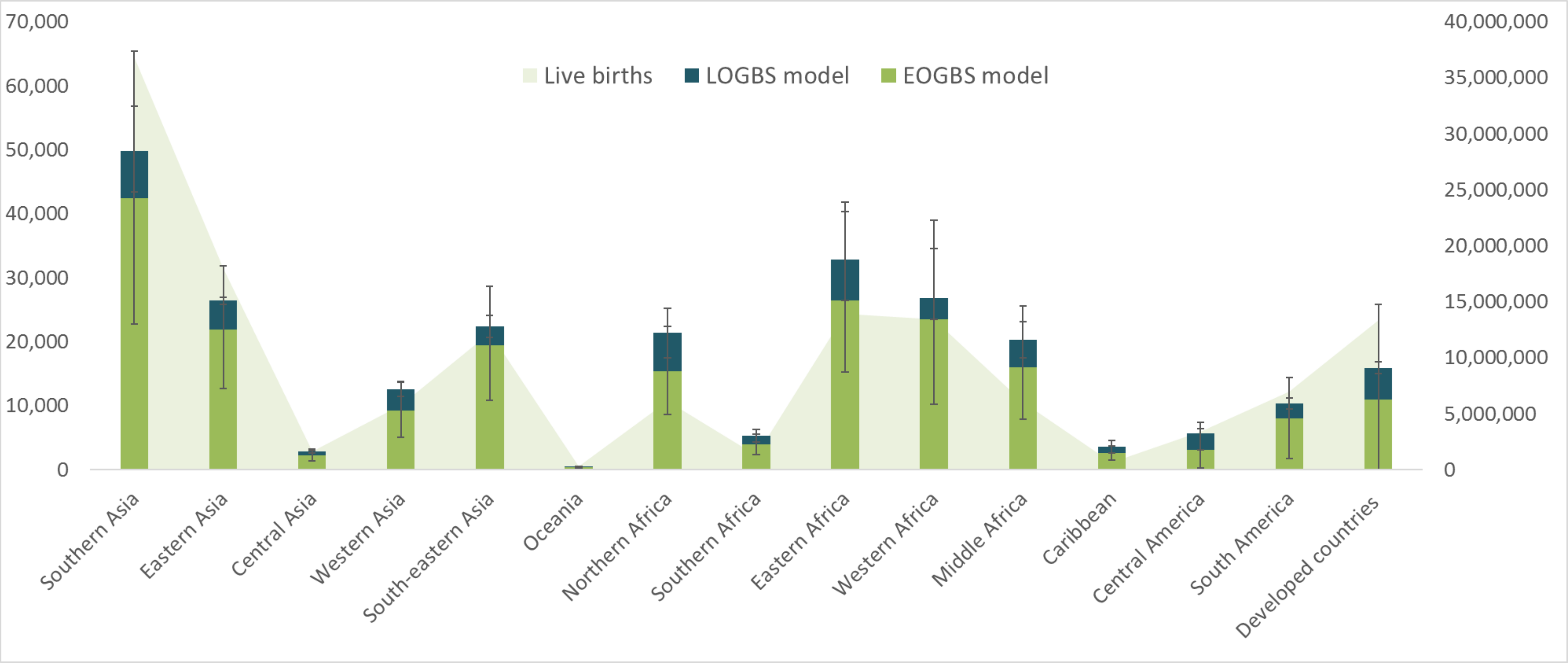

Supplementary Figure S5: Sensitivity analysis: Late onset infant Group B Streptococcal disease using a fixed ratio across regions for EOGBS:LOGBS, by sub-region for 2015.

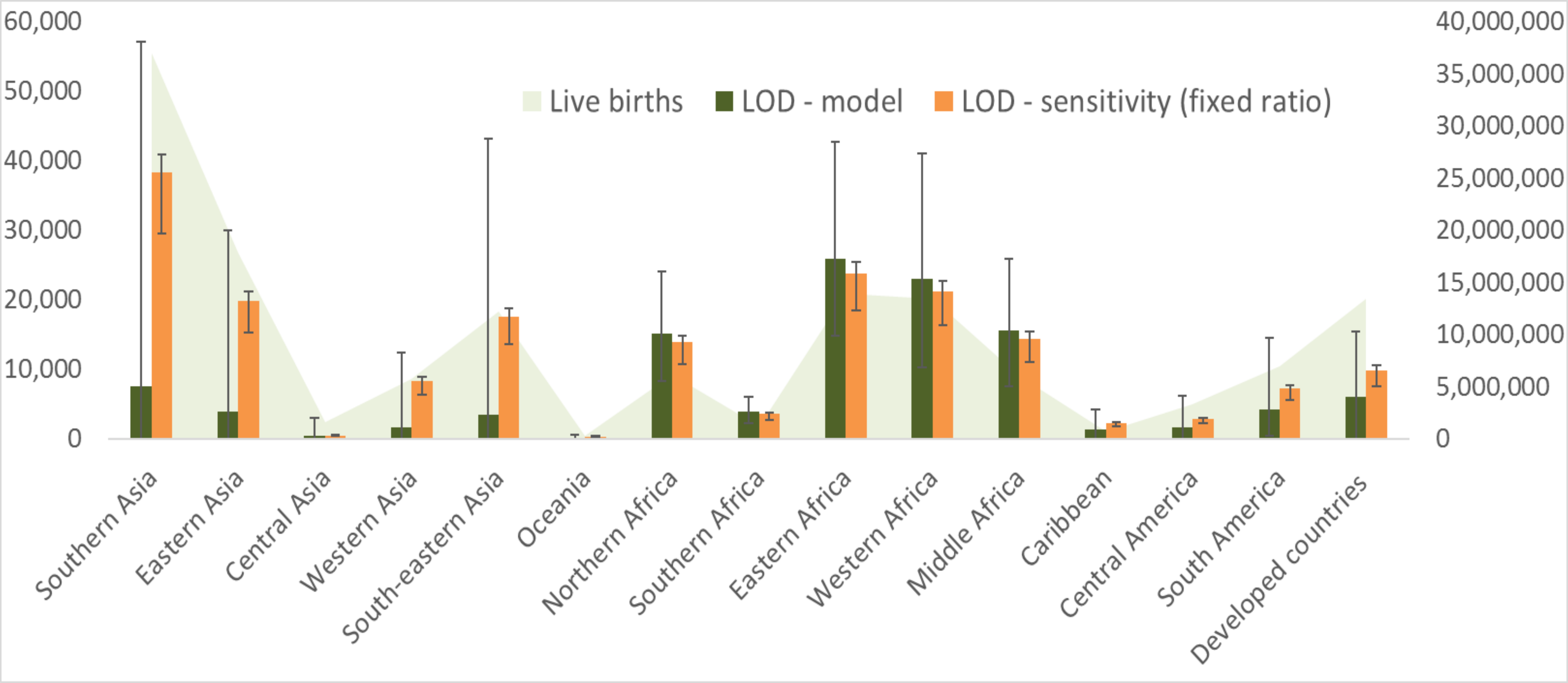

Supplementary Figure S6: Early onset and late onset Group B Streptococcal disease infant deaths, by sub-region for 2015.

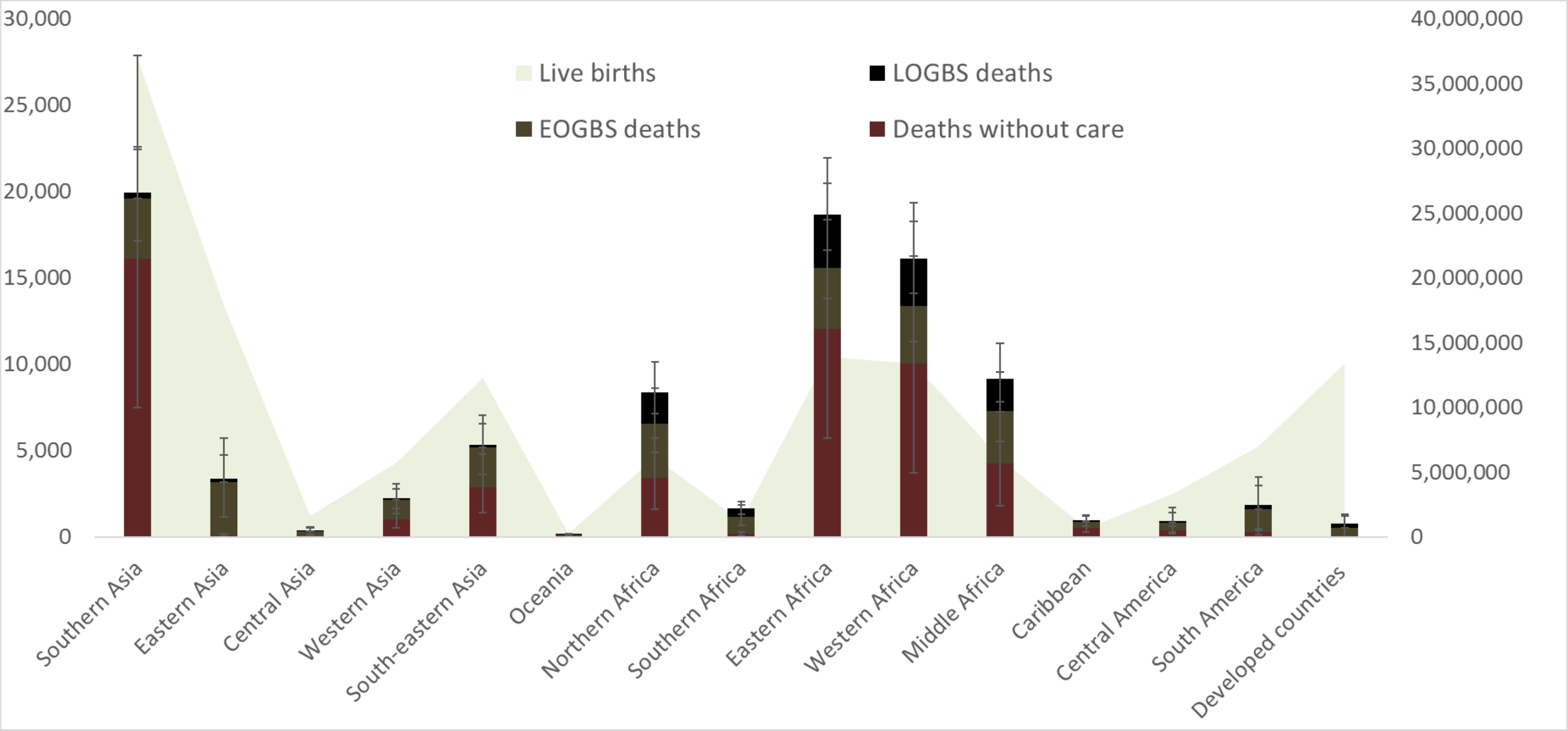

Supplementary Figure S7: Neurodevelopmental impairment (moderate-severe) after GBS meningitis, by sub-region for 2015

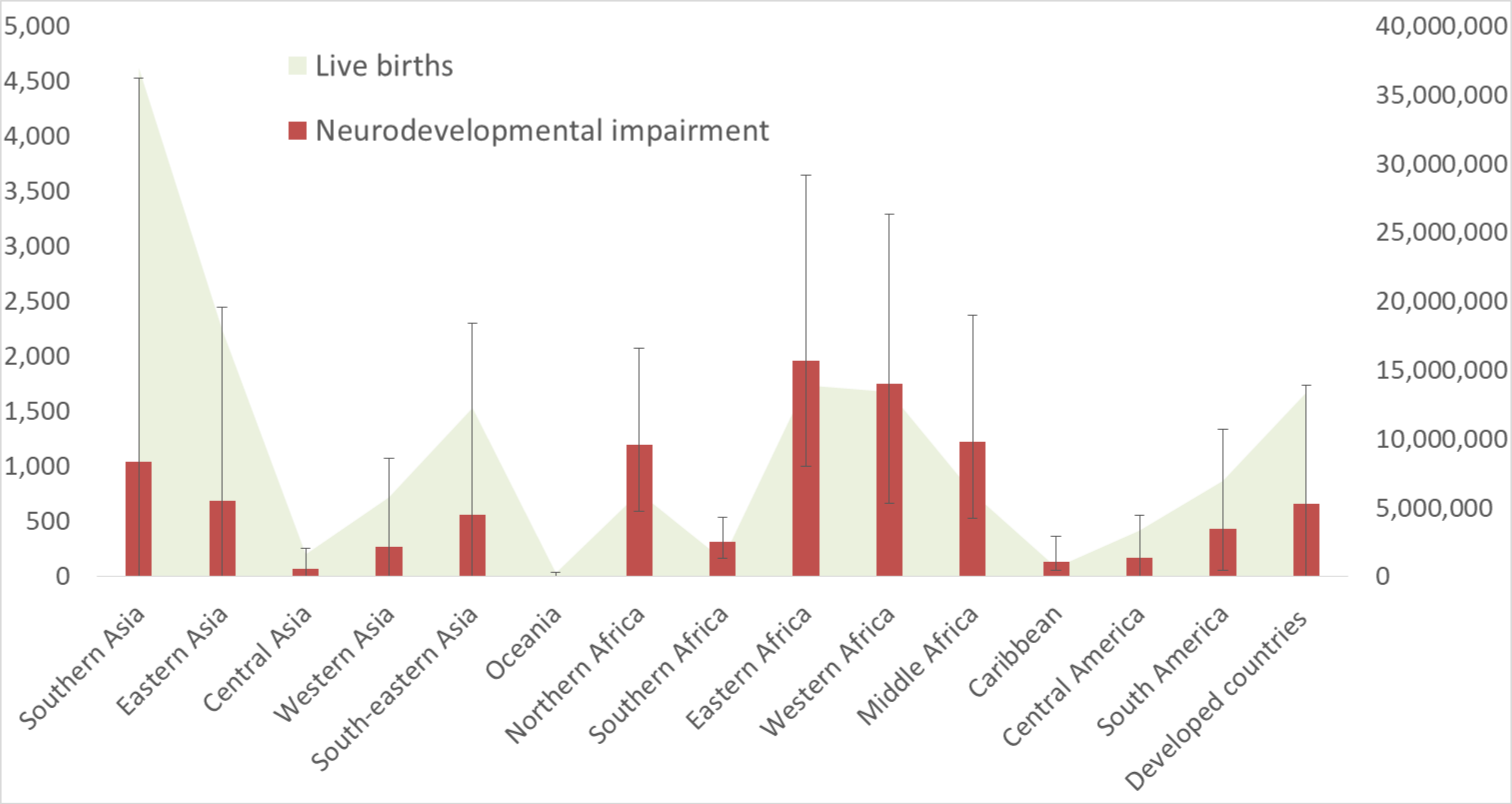

Supplementary Figure S8: Comparison of estimates of infants with EOGBS from compartmental model with incidence data, by sub-region for 2015

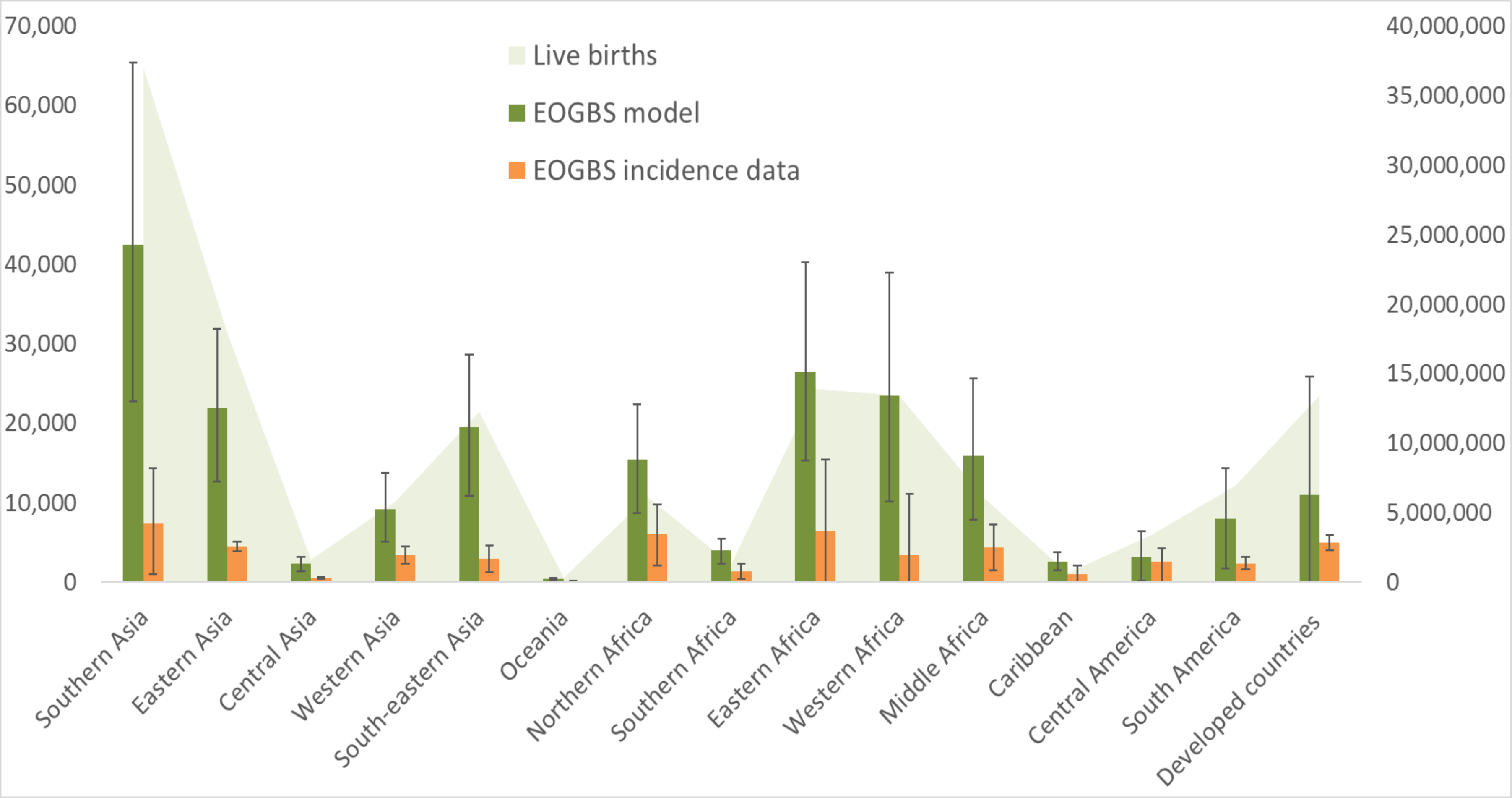

Supplementary Figure S9: Comparison of estimates of infants with LOGBS from compartmental model compared to incidence data, by sub-region for 2015

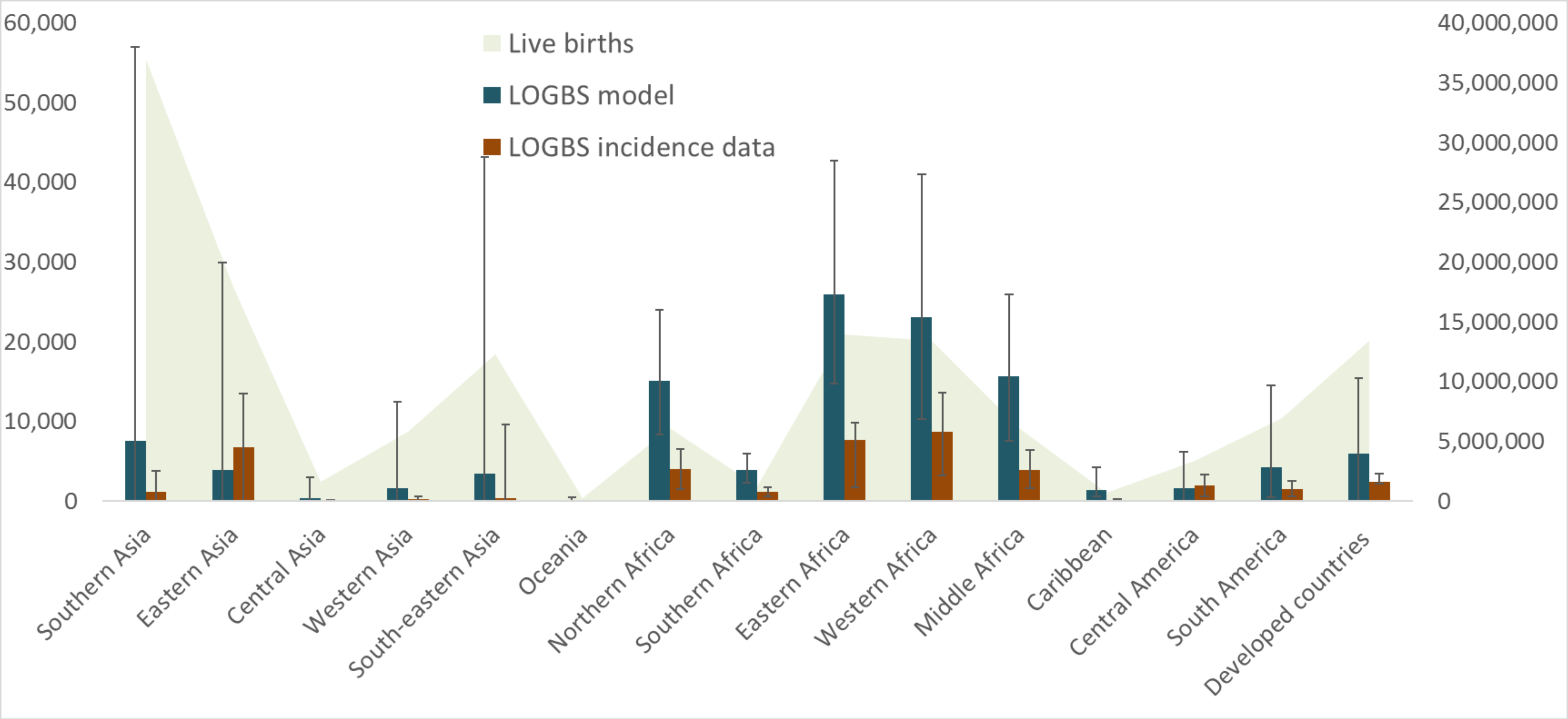

Supplementary Figure S10: Neonatal encephalopathy as a subset of infant invasive GBS disease cases, by sub-region for 2015

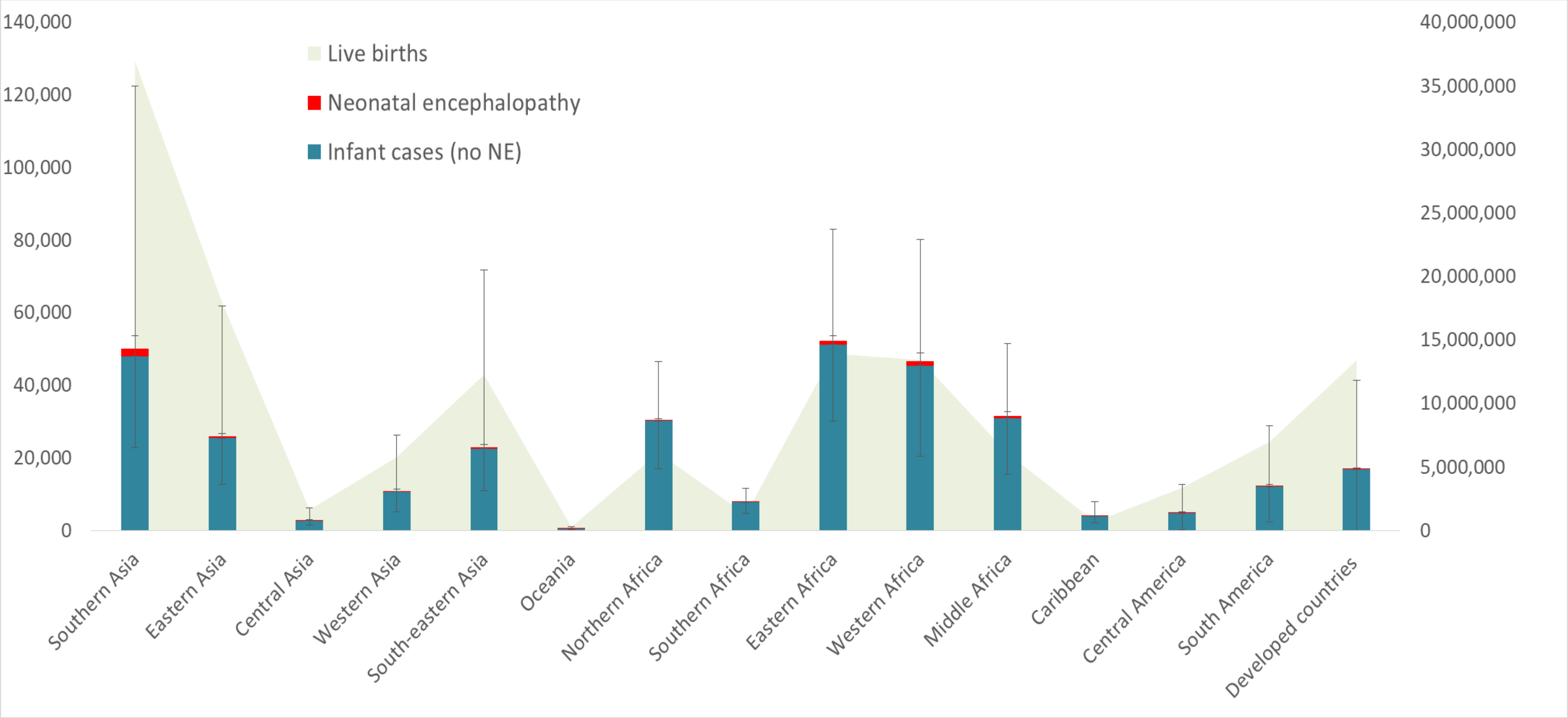

Supplementary Figure S11 Maternal GBS disease cases (minimum estimates), by sub-region for 2015

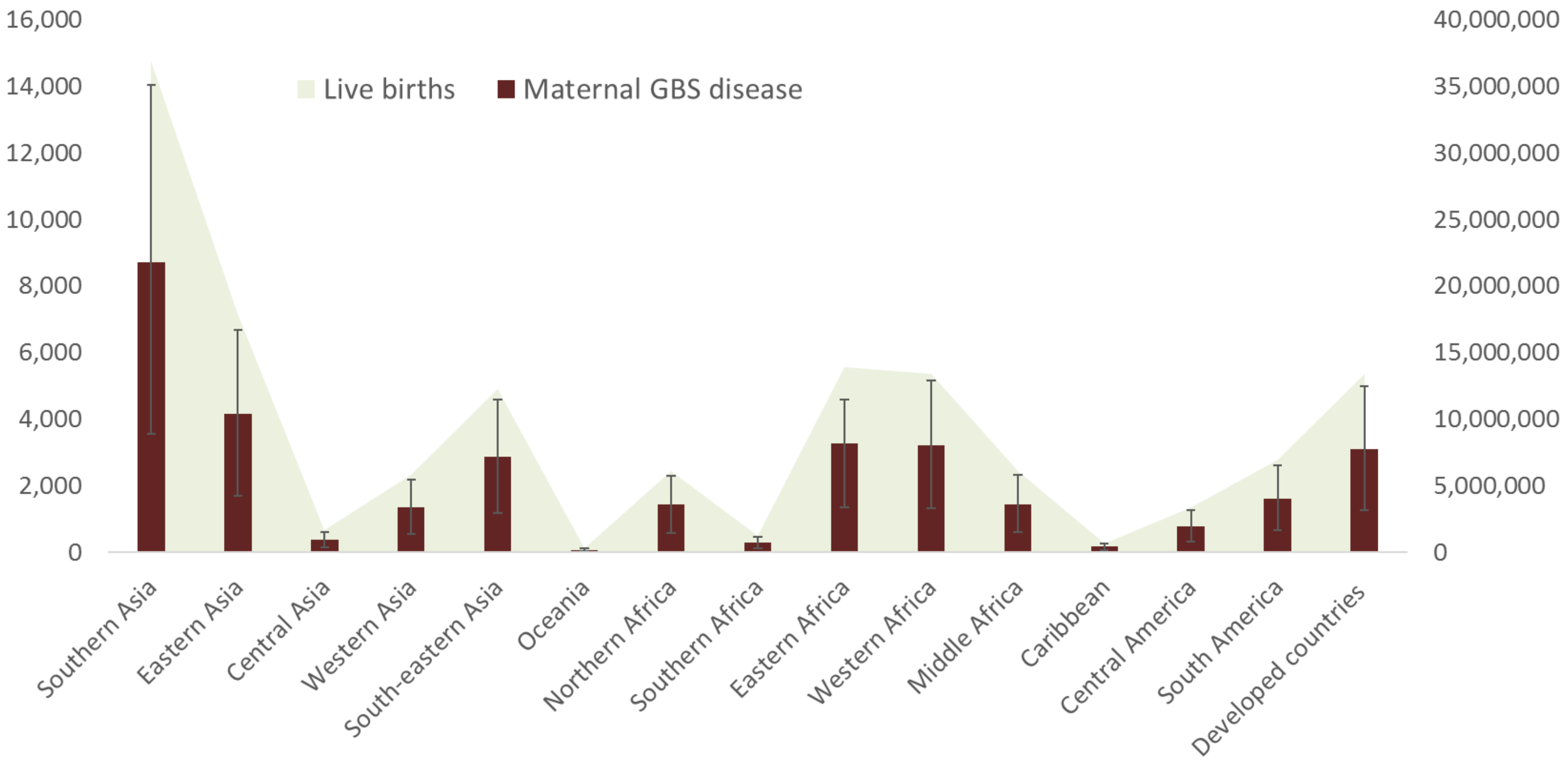

Supplementary Figure S12: Stillbirths with GBS disease, by sub-region for 2015

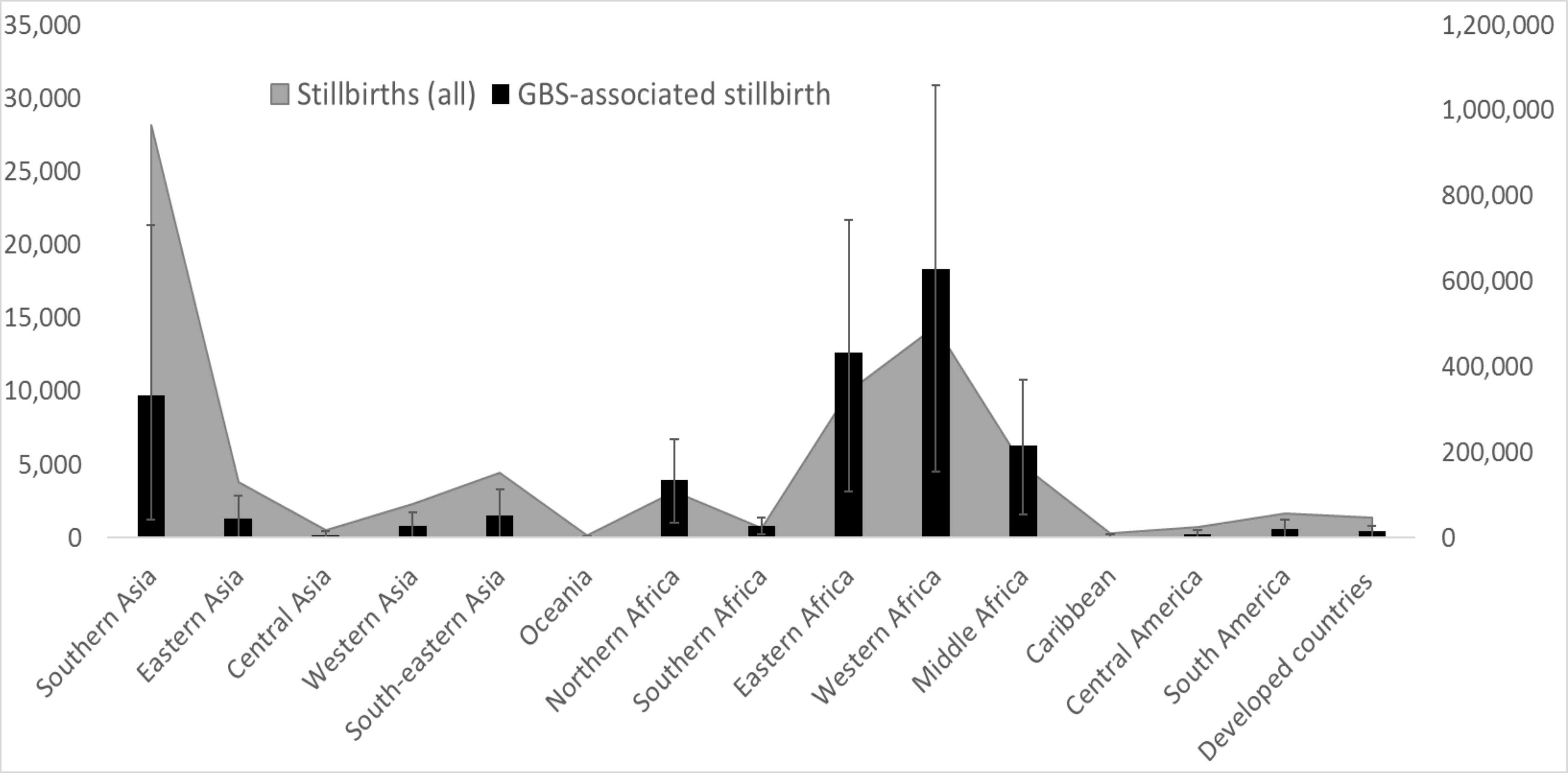

Supplementary Figure S13: Stillbirths with GBS disease, sensitivity analysis applying estimate from Africa to regions with no data by sub-region for 2015

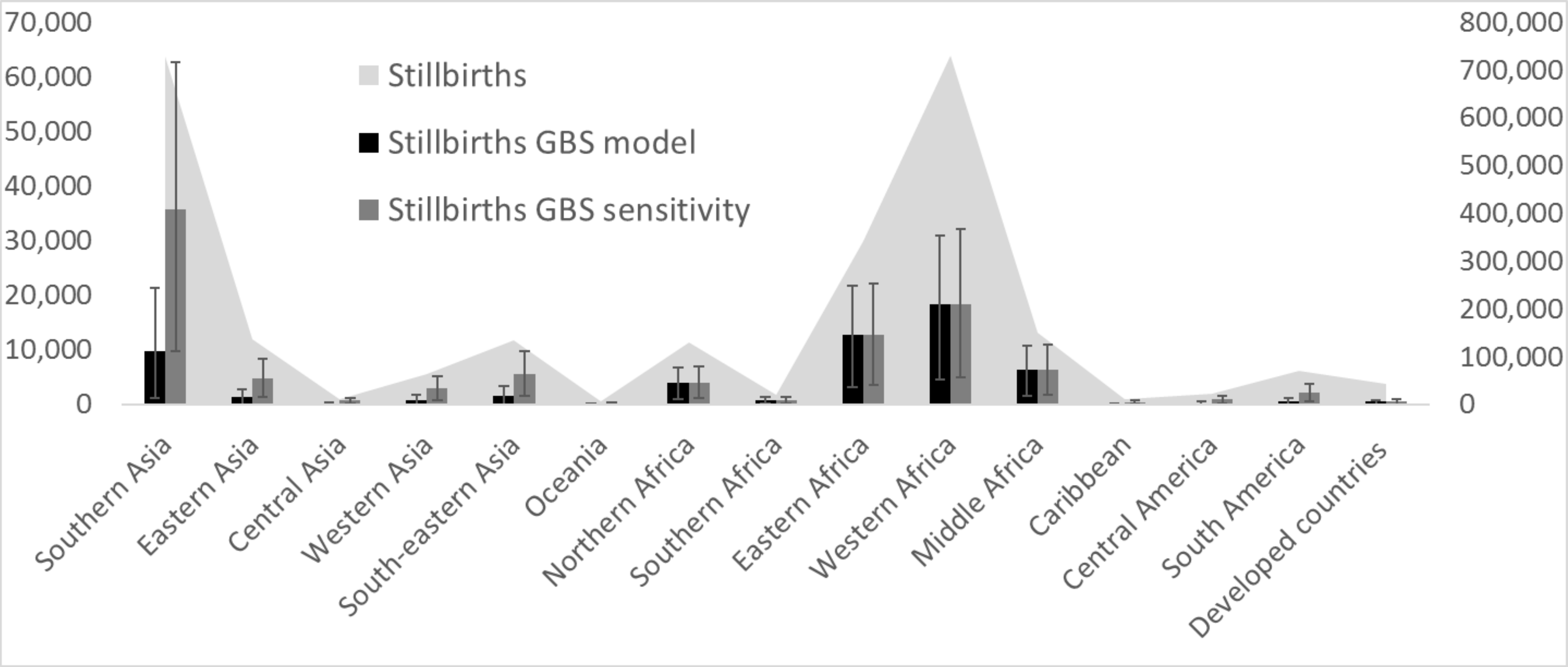

Supplement: Supplement-Material [file cix664_suppl_supplement-material.pdf]
